# The Use of Kosher Phenotyping for Mapping QTL Affecting Susceptibility to Bovine Respiratory Disease

**DOI:** 10.1371/journal.pone.0153423

**Published:** 2016-04-14

**Authors:** Ehud Lipkin, Maria Giuseppina Strillacci, Harel Eitam, Moran Yishay, Fausta Schiavini, Morris Soller, Alessandro Bagnato, Ariel Shabtay

**Affiliations:** 1 Department of Genetics, Hebrew University of Jerusalem, Jerusalem, Israel; 2 Università degli Studi di Milano (UNIMI), Milan, Italy; 3 Department of Ruminant Sciences, Agricultural Research Organization (ARO), Bet-Dagan, Israel; University of Florida, UNITED STATES

## Abstract

Bovine respiratory disease (BRD) is the leading cause of morbidity and mortality in feedlot cattle, caused by multiple pathogens that become more virulent in response to stress. As clinical signs often go undetected and various preventive strategies failed, identification of genes affecting BRD is essential for selection for resistance. Selective DNA pooling (SDP) was applied in a genome wide association study (GWAS) to map BRD QTLs in Israeli Holstein male calves. Kosher scoring of lung adhesions was used to allocate 122 and 62 animals to High (Glatt Kosher) and Low (Non-Kosher) resistant groups, respectively. Genotyping was performed using the Illumina BovineHD BeadChip according to the Infinium protocol. Moving average of -logP was used to map QTLs and Log drop was used to define their boundaries (QTLRs). The combined procedure was efficient for high resolution mapping. Nineteen QTLRs distributed over 13 autosomes were found, some overlapping previous studies. The QTLRs contain polymorphic functional and expression candidate genes to affect kosher status, with putative immunological and wound healing activities. Kosher phenotyping was shown to be a reliable means to map QTLs affecting BRD morbidity.

## Introduction

Bovine respiratory disease (BRD) complex is the leading world-wide cause of morbidity and mortality in feedlot cattle. It includes upper and lower respiratory tract infections, diphtheria and pneumonia [1,2]. Due to immature functionality of the respiratory system in young cattle [3], BRD occurs more frequently and severely at young age, regardless of immunological and management considerations [4,5]. BRD is the most costly feedlot disease due to prevention and treatment costs, morbidity, mortality, and production amortization that includes performance, carcass merit, meat tenderness and palatability [1,2,6,7].

BRD etiology is multifactorial, affected by a large number of stressors (e.g., weaning, transportation, commingling and others), and viral (*infectious bovine rhinotracheitis; IBR*, *bovine virus diarrhea; BVD*, *bovine respiratory syncytial virus; BRSV*) and bacterial pathogens (primarily, *Pasteurella multocida*, *Haemophilus somnus* and *Mycoplasma hyponeumoniae*). Many of these pathogens are normally present in the upper respiratory tract, but convert to pathogenic status as a consequence of stressful life history events [8]. Yet, individuals of the same age and environment, when exposed to BRD pathogens, vary greatly in whether they develop the disease [9], and the severity of clinical symptoms [10]. This variation suggests that genetic control is involved in susceptibility to BRD. Indeed, significant heritability for BRD resistance [11,12] and differences among breeds [11,13] have been documented.

During the years, various strategies have been implemented to prevent or minimize the prevalence of BRD. Among others, these include antibiotic treatment on a preventative or metaphylactic basis, non-antibiotic alternatives and vaccination [4]. Unfortunately, these strategies have collectively failed to reduce the prevalence of BRD (see citations in [14]). To date, methods for detecting morbid cattle involve subjective visual appraisal and depend on the stage and extent of the disease. However, clinical signs of BRD may often go undetected in feedlot calves [15], emphasizing the need of an objective and reliable early risk predictor [5]. Genomic-based approaches may thus serve as additional methods for control of BRD.

The importance of the genome in determining resistance and susceptibility to a wide variety of viral, bacterial and parasite-borne diseases is thoroughly documented [16,17]. Examples include mapping of quantitative trait loci (QTL) affecting trypanotolerance in the N’Dama cattle of West Africa [18], Marek’s Disease in layer chickens [19,20], and mastitis in Holstein dairy cattle [21]. With genome wide mapping and selection procedures based on high density SNP arrays [22], improving disease resistance through selection for resistance at the genome level has become realistic. Furthermore, high-resolution mapping of the QTLs responsible for genetic variation in resistance can serve as a platform to identify the causative genes themselves [23], opening further possibilities for disease control through greater understanding of the molecular mechanisms of resistance. For example, recent genome-wide association studies of Crohn’s Disease identified 71 loci associated with the disease [24] and new pathogenic mechanisms of the disease.

Reports on BRD QTL mapping are scarce. Using microsatellite markers, Neibergs *et al*. [25] identified QTLs affecting BRD on BTA 2 and 26 in a Brahman × Hereford sire half-sib family. Casas *et al*. [26] found association between BRD and SNPs in the *ANKRA2* and *CD180* genes on BTA 20 in a Brahman x Angus cross. Neibergs *et al*. [14] conducted genome-wide association study (GWAS) to map QTLs affecting BRD susceptibility in Holstein populations. Tizioto *et al*. [27] examined transcriptomes from bronchial lymph nodes of cattle challenged with three viral and three bacterial BRD pathogens, one at a time. Hundreds to thousands of genes changed expression in response to the pathogen challenge; 140 of which were located in previously mapped BRD QTLs.

To phenotypically distinguish between control and case individuals, Neibergs *et al*. [14] used common clinical signs of BRD, in combination with nasopharyngeal and pharyngeal recess swabs for the diagnosis of pathogens. An alternative well-accepted tool for retrospective diagnosis of BRD is lung lesions monitored at slaughter [28]. Among other categories, lung lesions include abscesses, parenchymal fibrosis, adhesions and emphysema [29]. Wittum *et al*. [15] found that damage resulting from BRD may leave persistent lesions in bovine lungs. Interestingly, lung lesions resulting from BRD are often found in animals never recorded for clinical BRD [2,6,15,30]. According to Gardner *et al*. [6], the high incidence of such cases reflected subclinical events, viral rather than bacterial infection, or disease occurrence at earlier stage, before calves were taken into feedlot. Still, pulmonary lesions at slaughter were indicative of BRD occurrence, which had a significant deleterious effect on production, independent of previous diagnosis of clinical illness [15]. In spite of the fact that it is the only practical way to detect subclinical BRD, only a few studies used lung lesions as an indicator of earlier life BRD episodes [4].

As mentioned above, Bryant *et al*. [29] classified lung adhesions in the cranioventral lobes as a specific example of lung lesions. Adhesions are formed as a normal part of the body's healing process and help to limit the spread of infection. They are fibrous bands of scar tissue that span the pleural space, between the parietal and visceral layers of the pleura and often between the lobes of the lungs, or between the lungs and the rib cage, and are caused by repeated episodes of inflammation of the lungs. The fibrin bands may eventually dissolve through fibrinolysis, and the traumatized site continues to heal, but there are cases in which fibrinolysis is inhibited [31]. Thus, uncontrolled fibrosis may render the repair process pathogenic, resulting in excess deposition of extracellular matrix (ECM) components, including collagen, and replacement of normal tissue by permanent scar tissue, which influences organ function [32]. In a recent report [33], calves free of pulmonary adhesions at slaughter were found biologically and economically more efficient than their affected peers. Interestingly, pleural adhesions found in ca. 40% of the calves accounted for reduced growth rate at early life stages [2].

Kosher slaughtering, a routine procedure in Israel and other countries [34], offers particular advantages for genetic analysis of BRD. Kosher slaughtering involves two steps: The actual slaughter, and then a close and detailed examination of the lungs of the slaughtered animals for adhesions. Cattle presenting lungs completely clear of adhesions, indicative of their having been BRD free, are classed as “Glatt” kosher. Cattle presenting severe pulmonary adhesions, indicative of one or more severe bouts of BRD, are classed as “non-kosher” or "treif". Animals presenting light adhesions are classed as standard-kosher [35].

In the current study, we mapped BRD QTLs in Israeli Holstein male calves by means of selective DNA pooling (SDP), using the kosher slaughtering three-level classification system to distinguish between High (Glatt) and Low (Treif) resistant phenotypes, Numerous QTLs were found and their surrounding regions (QTLRs) searched for candidate genes and for amino acid (AA) polymorphisms within these genes. The candidate genes involved immunological response and wound healing activities that include cell adhesion, extra cellular matrix (ECM) remodeling, epithelial-to-mesenchyme transition, and profibrotic responses. The QTLRs and their genes partially overlap previous QTLs mapping studies, supporting the use of kosher phenotype for BRD QTL mapping.

## Results

### Markers

Animals were allocated to High and Low resistant groups by a kosher inspection of lung adhesions. Frequencies of allele B in the pools were obtained by Illumina software. Frequency differences between High and Low groups (D_i_) and an empirical estimate of the standard error of D_i_, were used to obtain Comparison-wise-Type I error rate (P-values). The P-values over all autosomes are presented in S1 Fig. Table 1 shows critical marker P-values required to achieve significance at the given PFP thresholds. A total of 749 markers were in the range of PFP ≤ 0.2, distributed over all autosomes. Of course, the number of QTLs is much less than the numbers of significant markers, as many significant markers are associated with the same QTL. The estimated number of falsified and true null hypotheses, n_1_ and n_2_ [36,37] were 2,114 and 568,449 respectively. Thus, effective power at PFP ≤ 0.2 was 0.35.

**Table 1:** Critical P-values and number of significant SNPs, by PFP level.

PFP	Critical P	Sig SNPs
< 0.01	1.63E-06	95
> 0.01–0.05	2.06E-05	148
> 0.05–0.10	6.91E-05	152
> 0.10–0.20	2.64E-04	354
> 0.20–0.40	1.02E-03	712

### QTLRs detection

**Fig 1:**
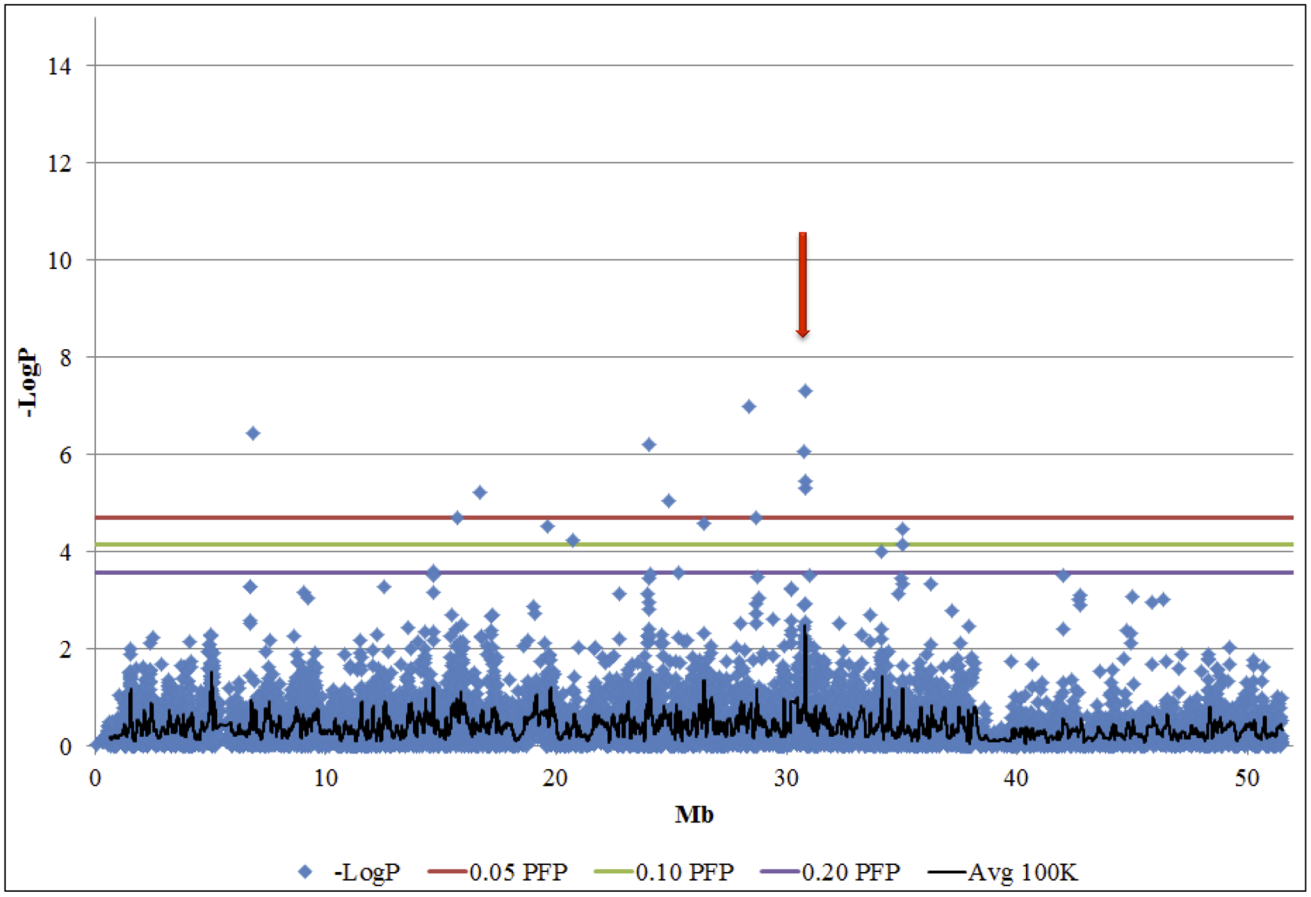
A cluster of significant -LogP values on BTA 29 at about 30 Mb (red arrow; Fig 2 and Table 2). Blue diamonds, -LogP values of the markers; Avg 100K, moving average -LogP values of windows of 23 markers (≈ 100Kb; see text). Note that for this cluster the peak value of the moving average exceeds the -LogP = 2.0 threshold chosen to declare significance.

**Fig 2:**
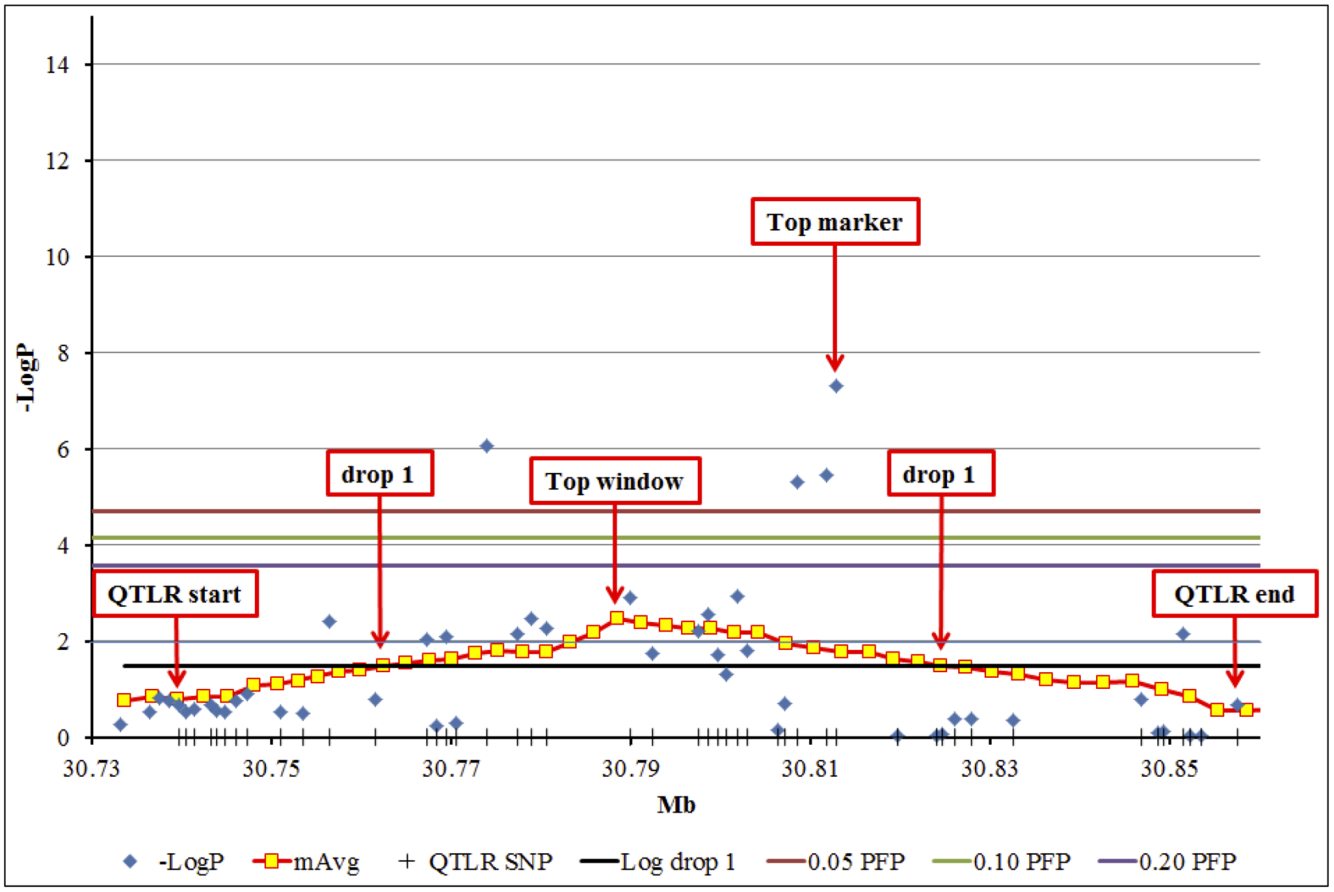
Expanded view of the QTLR at 30 Mb on BTA 29 (Fig 1 and Table 2). Vertical bars on the X-axis, QTLR marker locations; Blue diamonds, -LogP values of the markers; Yellow squares: X-axis, mean location of the markers in the window; Y-axis, mean -LogP of the window. QTLR start and end, up- and down-stream boundary markers of the QTLR; Drop 1, up and down-stream Log drop 1 boundary windows; Top window, the window with highest average -LogP; Top marker, the most significant marker of the cluster. Three uppermost horizontal bars from top down: significance thresholds for individual markers at PFP = 0.05, 0.10 and 0.20, respectively. Two lowest horizontal bars, from top down: significance threshold for moving average of -LogP = 2.0; Log drop 1 threshold (from Peak window), respectively.

Visual inspection of chromosomal scatter charts (S1 Fig) revealed clusters of significant marker -LogP values (e.g., Fig 1). We considered such clusters to represent QTLRs. Detailed examination of the makers in the QTLR showed that they were a mixture of significant markers and non-significant markers, making it difficult to set boundaries for the QTLR. To circumvent this, we used a moving average of marker -LogP values with a window size of 23 markers (about 100 kb), and a Log drop of 1 to define QTLRs and their boundaries (details in Methods). This worked well and clear peaks with monotonic shoulders were obtained. A moving average of -LogP ≥ 2, corresponding to P = 0.01, was set as the threshold value for declaration of a window as a QTLR, irrespective of whether the window contained a significant marker or not. Fig 2 presents a detailed example of defining a QTLR based on the cluster on BTA 29 (Fig 1).

**Fig 3:**
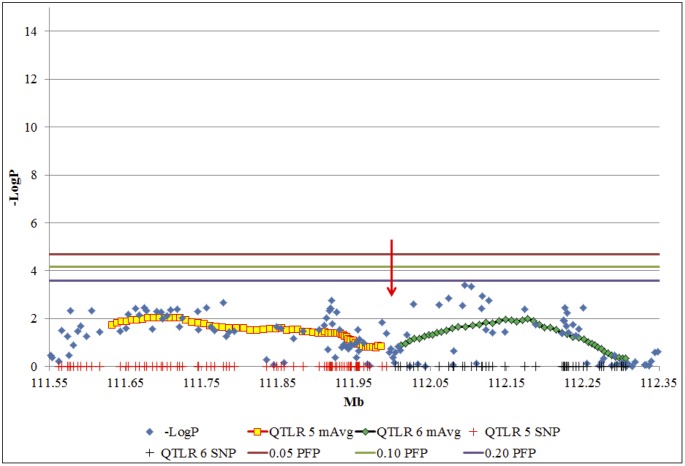
Example of a region with two mapped QTLRs that are possibly only one putative QTLR. QTLRs 5 and 6 on BTA 2. Red and black vertical bars on the X-axis, QTLRs 5 and 6 marker locations; Blue diamonds, -LogP values of the markers; Yellow squares (QTLR 5) and green diamonds (QTLR 6): X-axis, mean location of the markers in the window; Y-axis, mean -LogP of the window; red arrow, inter QTLR cluster. Three uppermost horizontal bars from top down: significance thresholds for individual markers at PFP = 0.05, 0.10 and 0.20, respectively.

Based on our criteria for declaring a QTLR, a total of 19 QTLs were found, distributed over 13 chromosomes (Table 2). Of these, 16 represent clearly distinguished clusters of significant markers, while three represent windows that reached the -LogP < 2 criterion, but did not contain significant markers. They were, however, characterized by a stretch of high value -LogP values, without admixture of low -LogP value markers. Although declared as individual QTLs on our Log drop 1 criterion, the two closely linked QTLRs 5 and 6 on BTA 2 may possibly represent only a single QTLR (Fig 3). The two QTLR cover the region of 111.5–112.3 Mb on BTA 2. Close inspection reveals an intermediate cluster of low -LogP values, around 112.0 Mb (red arrow in Fig 3). It was this low cluster that dropped the -LogP of the intermediate windows below the Log drop 1 of both sides, and thus split this region into two QTLR.

**Table 2:** Autosomal QTLRs.

QTLRs							Avg of top window^d^		Top SNP^e^		
QTLR	BTA	Start^a^	End^b^	Length	Distance^c^	SNPs	bp	P	Name	bp	P
1	1	32,762,621	33,000,982	238,362		49	32,922,068	7.2x10^-2^	rs43229554	32,892,280	2.7x10^-15^
2	1	92,104,370	92,235,747	131,378	59,103,388	46	92,172,725	2.8x10^-1^	rs42857937	92,194,011	9.8x10^-10^
3	1	136,141,849	136,617,538	475,690	43,906,102	101	136,234,581	8.3x10^-2^	rs109727900	136,269,109	2.6x10^-5^
4	2	103,578,039	103,858,856	280,818		71	103,604,186	7.4x10^-2^	rs110744763	103,622,473	7.4x10^-6^
5	2	111,562,742	111,992,526	429,785	7,703,886	73	111,702,216	1.3x10^-2^	rs41718804	111,920,205	1.7x10^-3^
6	2	112,003,692	112,306,444	302,753	11,166	46	112,177,753	4.7x10^-2^	rs109625954	112,094,944	3.9x10^-4^
7	2	114,545,480	114,830,722	285,243	2,239,036	50	114,692,948	1.1x10^-1^	rs42619825	114,679,293	1.4x10^-7^
8	8	41,810,125	42,014,357	204,233		47	41,951,028	2.5x10^-1^	rs109097634	41,958,832	1.6x10^-6^
9	9	103,591,751	103,738,680	146,930		64	103,644,910	6.1x10^-3^	rs109603023	103,658,874	7.2x10^-8^
10	10	55,539,090	55,719,332	180,243		57	55,606,200	1.7x10^-2^	rs43633836	55,591,993	2.3x10^-3^
11	12	7,221,507	7,469,740	248,234		53	7,357,534	1.3x10^-2^	rs134347273	7,306,510	8.9x10^-5^
12	15	2,491,081	2,679,720	188,640		45	2,593,214	4.5x10^-3^	rs132966783	2,560,990	8.1x10^-5^
13	15	35,995,220	36,429,977	434,758	33,315,500	72	36,183,585	2.1x10^-2^	rs110068780	36,198,691	3.7x10^-5^
14	16	38,146,214	38,575,149	428,936		97	38,370,212	2.0x10^-2^	rs136111126	38,382,048	2.0x10^-5^
15	18	58,445,044	58,667,459	222,416		44	58,614,523	1.4x10^-1^	rs43073607	58,634,645	2.1x10^-5^
16	22	55,739,008	55,889,024	150,017		47	55,804,668	3.9x10^-2^	rs135721055	55,798,814	5.2x10^-5^
17	24	26,944,578	27,174,025	229,448		56	27,068,708	1.2x10^-1^	rs134945287	27,080,758	2.8x10^-5^
18	26	43,067,146	43,222,533	155,388		46	43,172,648	8.4x10^-2^	rs132928018	43,178,710	1.6x10^-5^
19	29	30,739,666	30,860,467	120,802		47	30,788,390	1.0x10^-1^	rs134937987	30,812,830	5.0x10^-8^

^a^First marker of the first significant window.

^b^Last (23^rd^) marker of the last significant window.

^c^The distance between the present and the previous QTLR on the same chromosome = the length between the end and the start of the up- and down- stream QTLRs.

^d^The window with highest -LogP value.

^e^Most significant SNP in the QTLR.

**Fig 4:**
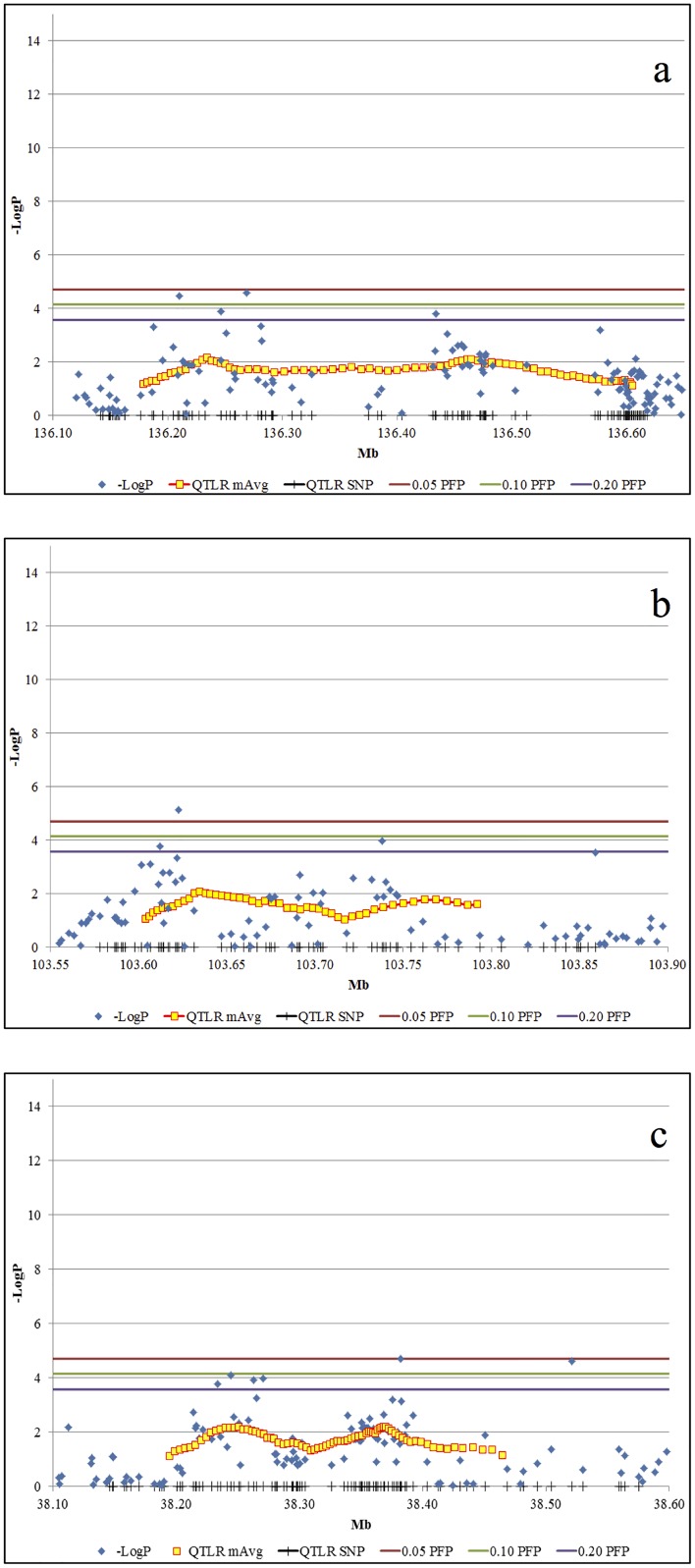
Examples of chromosomal regions with one QTLR but possibly including two putative QTLRs. a. QTLR 3 on BTA 1. b. QTLR 4 on BTA 2. c. QTLR 14 on BTA 16. Vertical bars on the X-axis, QTLR markers locations; Blue diamonds, -LogP values of the markers; Yellow squares: X-axis, mean location of the markers in the window; Y-axis, mean -LogP of the window. Three uppermost horizontal bars from top down: significance thresholds for individual markers at PFP = 0.05, 0.10 and 0.20, respectively.

Conversely, QTLR 3, 4, and 14, each present two separated peaks but the intermediate regions did not meet our Log drop criteria for declaration as separate QTLRs. Thus, they may possibly represent a closely linked pair of QTLR each (Fig 4).

The QTLRs averaged 58 markers (range 44–101 markers), 255,478 bp (range 120,802–475,690 bp), covering a total of 4,854,074 bp, 0.19% of total of the 2.6 Gb bovine genome.

### Genes in the QTLR

The 19 QTLRs (Table 2) were composed of a total of 1,111 array markers (S1 Table, SNP report sheet). Based on public databases, all except one of these array marker SNPs were non-coding. These regions and markers were used for detailed bioinformatics analyses. Among the 1,111 markers, 545 mapped within 35 genes (Table 3). KCNE4 in QTLR 5 on BTA 2 is also presented in Table 3, even though no SNP was mapped to it. Thus, Table 3 presents a total of 36 genes. Among these, annotation data were available in David Database for only 20 genes.

**Table 3:** Complete list of all genes within the QTLRs or within 0.5 Mb upstream or downstream of the QTLR boundaries.

QTLR		Gene			Intergenic SNPs No.^c^
No.	BTA	Name/Flank	SNPs		
			No.^a^	P<0.01^b^	
**1**	**1**	Upstream	*CADM2*		
		*CADM2*	49	10	0
		Downstream	*CADM2*		
**2**	**1**	Upstream	*NAALADL2*		
		*NAALADL2*	46	8	0
		Downstream	*NAALADL2*		
**3**	**1**	Upstream	*EPHB1*, *KY*, *CEP63*, *ANAPC13*, *AMOTL2*		
		*RYK*	26	11	25
		*SLCO2A1*	21	9	
		*RAB6B*	29	2	
		Downstream	*RAB6B*, *SRPRB*, *TF*, *LOC525947*, *TOPBP1*, *CDV3*, *BFSP2*, *TMEM108*		
**4**	**2**	Upstream	*VWC2L*, *BARD1*, *ABCA12*		
		*ABCA12*	46	14	20
		*ATIC*	5	1	
		Downstream	*ATIC*, *FN1*, *MIR2285L*		
**5**	**2**	Upstream	*PAX3*, *SGPP2*		
		*FARSB*	5	1	58
		*LOC538702*	1	0	
		*MOGAT1*	2	1	
		*ACSL3*	7	0	
		*KCNE4*	0		
		Downstream	-		
**6**	**2**	Upstream	*FARSB*, *LOC538702*, *MOGAT1*, *ACSL3*, *KCNE4*		
		-	0		46
		Downstream	*AP1S3*, *SCG2*, *WDFY1*		
**7**	**2**	Upstream	-		
		*NYAP2*	16	0	34
		Downstream	*LOC104969998*		
**8**	**8**	Upstream	*RFX3*, *LOC101905621*, *LOC101905770*		
		*KIAA0020*	18	9	29
		Downstream	*KCNV2*, *VLDLR*		
**9**	**9**	Upstream	*RPS6KA2*, *RNASET2*, *FGFR1OP*, *CCR6*, *GPR31*, *TTLL2*, *UNC93A*		
		*LOC101905262*	5	0	59
		Downstream	*MLLT4*, *KIF25*, *FRMD1*, *DACT2*, *SMOC2*		
**10**	**10**	Upstream	*PIGB*, *RAB27A*, *RSL24D1*, *SNORA25*		
		*UNC13C*	1	0	56
		Downstream	-		
**11**	**12**	Upstream	-		
		-	0		53
		Downstream	-		
**12**	**15**	Upstream	*MSANTD4*		
		*GRIA4*	45	20	0
		Downstream	-		
**13**	**15**	Upstream	*OTOG*, *USH1C*, *ABCC8*, *KCNJ11*, *NCR3LG1*, *NUCB2*, *PI3KC2a*, *RPS13*		
		*PLEKHA7*	36	14	23
		*C15H11orf58*	5	1	
		*SOX6*	8	0	
		Downstream	-		
**14**	**16**	Upstream	*NME7*, *BLZF1*, *CCDC181*, *SLC19A2*, *F5*, *SELP*, *SELL*		
		*SELL*	6	0	24
		*SELE*	16	5	
		*C16H1orf112*	16	4	
		*SCYL3*	5	0	
		*KIFAP3*	29	9	
		*METTL11B*	1	0	
		Downstream	*GORAB*, *PRRX1*		
**15**	**18**	Upstream	*SIGLEC5*, *MIR99B*, *MIRLET7E*, *MIR125A*, *HAS1*, *VN2R408P*, *ZNF613*, *ZNF615*, *ZNF614*, *ZNF432*, *ZNF350*		
		*PPP2R1A*	1	0	33
		*BOSTAUV1R416*	1	0	
		*BOSTAUV1R417*	4	0	
		*ZNF415*	5	0	
		Downstream	*LOC539675*, *LOC787057*, *LOC100848895*, *LOC506495*, *LOC785630*		
**16**	**22**	Upstream	*SLC6A11*, *SLC6A1*		
		*HRH1*	13	0	34
		Downstream	*ATG7*, *VGLL4*, *TAMM41*		
**17**	**24**	Upstream	*DSC3*		
		-	0	0	56
		Downstream	-		
**18**	**26**	Upstream	*PLEKHA1*, *HTRA1*, *DMBT1*, *SPADH1*, *SPADH2*		
		*C26H10orf88*	1	0	16
		*PSTK*	1	0	
		*IKZF5*	5	0	
		*ACADSB*	23	15	
		Downstream	*HMX3*, *HMX2*, *BUB3*		
**19**	**29**	Upstream	*KIRREL3*		
		*KIRREL3*	47	15	0
		Downstream	*KIRREL3*, *LOC104976256*		

^a^Number of SNPs within the gene;

^b^Number of SNPs with P ≤ 0.01 within the gene.

^c^Number of SNPs in the QTLR in the regions between genes.

Considering all genes included within or in the 0.5 Mb flanks of the QTLRs, a total of 130 genes were found, of which annotation data were available for 98. Their biological processes (BP), cellular components (CC), molecular function (MF) and metabolic pathways (KEGG) are detailed in S1 Table (‘gene not clustered’ and ‘Kegg pathway’ sheets, respectively). Among these 98 genes, we focused on 18 candidates, based on biological and statistical considerations as detailed in the Discussion. We searched NCBI dbSNP for non-synonymous (AA substitution) polymorphisms, candidates to be the causative mutation of the found BRD effect. The NCBI dbSNP present a substantial polymorphism in all 18 genes (Table 4). These genes averaged 664.5 AA, 1,993.5 bp. All genes had SNPs in the coding regions (average 7.8% of all SNPs); all had AA substitutions (average 78.4% of the coding SNPs) and all had AA substitutions involving change of properties (average 60.4% of all AA substitutions). Although these are published polymorphisms, not polymorphisms found in the study population, they present the potential functional polymorphisms of the QTLR genes.

**Table 4:** Non-synonymous polymorphisms in the selected 18 candidate genes as reported in NCBI dbSNP.

					SNPs^c^		Non-synon.^d^		Properties change^e^	
QTLR	BTA	Gene	AA^a^	Bp^b^	No.	Prop.	No.	Prop.	No.	Prop.
1	1	CADM2	444	1,332	52	0.039	39	0.750	19	0.487
2	1	NAALADL2	882	2,646	156	0.059	130	0.833	78	0.600
3	1	SLCO2A1	644	1,932	98	0.051	72	0.735	37	0.514
3	1	TF	703	2,109	113	0.054	88	0.779	60	0.682
4	2	ABCA12	2,593	7,779	297	0.038	215	0.724	132	0.614
6	2	AP1S3	154	462	15	0.032	12	0.800	8	0.667
8	8	KIAA0020	647	1,941	136	0.070	105	0.772	68	0.648
9	9	SMOC2	445	1,335	214	0.160	196	0.916	115	0.587
9	9	CCR6	375	1,125	75	0.067	53	0.707	32	0.604
10	10	RAB27A	221	663	59	0.089	43	0.729	23	0.535
13	15	PLEKHA7	1,224	3,672	605	0.165	505	0.835	307	0.608
14	16	SELE	485	1,455	46	0.032	37	0.804	22	0.595
14	16	SELP	646	1,938	43	0.022	31	0.721	20	0.645
14	16	SELL	370	1,110	30	0.027	24	0.800	17	0.708
15	18	PPP2R1A	589	1,767	443	0.251	383	0.865	207	0.540
16	22	HRH1	491	1,473	191	0.130	158	0.827	94	0.595
16	22	ATG7	629	1,887	151	0.080	117	0.775	53	0.453
18	26	IKZF5	419	1,257	60	0.048	45	0.750	36	0.800
		Avg	664.5	1,993.5	154.7	0.078	125.2	0.784	73.8	0.604
		Min	154	462	15	0.022	12	0.707	8	0.453
		Max	2,593	7,779	605	0.251	505	0.916	307	0.800

^a^Number of amino acid.

^b^Number of coding nucleotides in the gene.

^c^No. and Prop.: number of coding SNPs and their proportion out of all coding nucleotides.

^d^No. and Prop.: number of non- synonymous SNPs and their proportion out of all SNPs.

^e^No. and Prop.: number of AA substitution involving change of AA physical properties, and their proportion out of all non-synonymous SNPs).

## Discussion

BRD is the most prevalent disease in the cattle industry in many parts of the world, with symptoms that often go undetected. Using the kosher phenotypic classification we performed a genome-wide association study (GWAS) to map QTL affecting BRD morbidity in Israeli Holstein calves. SDP and Illumina BovineHD BeadChip were used to scan the genome.

Typically, significant markers found by a GWAS are intermingled with non-significant markers (S1 Fig). The lack of a monotonic relation between marker location and marker significance makes it impossible to apply the widely used LOD drop method to set boundaries for the QTLR. To circumvent this we used a moving average of -LogP. This worked well, and clear peaks with monotonic shoulders were obtained (Fig 2). Using a threshold of moving average of -LogP = 2 (corresponding to P = 0.01) to identify QTLs yielded 19 QTLRs distributed over 13 autosomes (Table 2).

The commonly used Log drop 1 method was effective in setting clear boundaries for the QTLR, in one case distinguishing between QTLR as close as 11 Kb (between QTLRs 5 and 6; Table 2 and Fig 2). While distinguishing between two very close QTL on BTA 2 (Fig 3), it nevertheless merged possibly distinct QTL on BTAs 1, 2 and 16 (Fig 4).

Among the highly significant markers (with P < 0.001) in the QTLRs, 48.1% were generated by markers with MAF < 0.15 (S1 Table). This high proportion could be a result of an underestimation of allele frequency variance for markers with low MAF. However, in this study P-values were obtained by SD adjusted to allele B frequency (see Materials and Methods). The distribution of the SD values against allele B frequency showed no indication of a secondary mode at the high SD levels that would indicate presence of an appreciable group of unstable markers (data not shown). We are not aware of any report on the distribution to compare with of MAF in QTLRs. The high proportion of low MAFs among highly significant QTLRs markers could be a result of recent new mutations, selection, or both. This, however, is beyond the scope of the present study.

Combining moving average with Log drop resulted in high resolution mapping, with average QTLR size of 0.26 Mb, allowing thorough bioinformatic analysis to identify candidate genes in the proximity of those QTLR. Numerous databases and analyses were used to locate and categorize genes in and around the 19 QTLRs. Among the genes within or in the 0.5 Mb flanks of the QTLRs (Table 3), we focused on 18 candidate gene, based on biological and statistical considerations (Table 4). These included involvement of the genes in immunity, wound repair, cells adhesion and pulmonary function; the number of significant SNPs within the gene; and proximity of previously reported relevant QTLRs.

**Fig 5:**
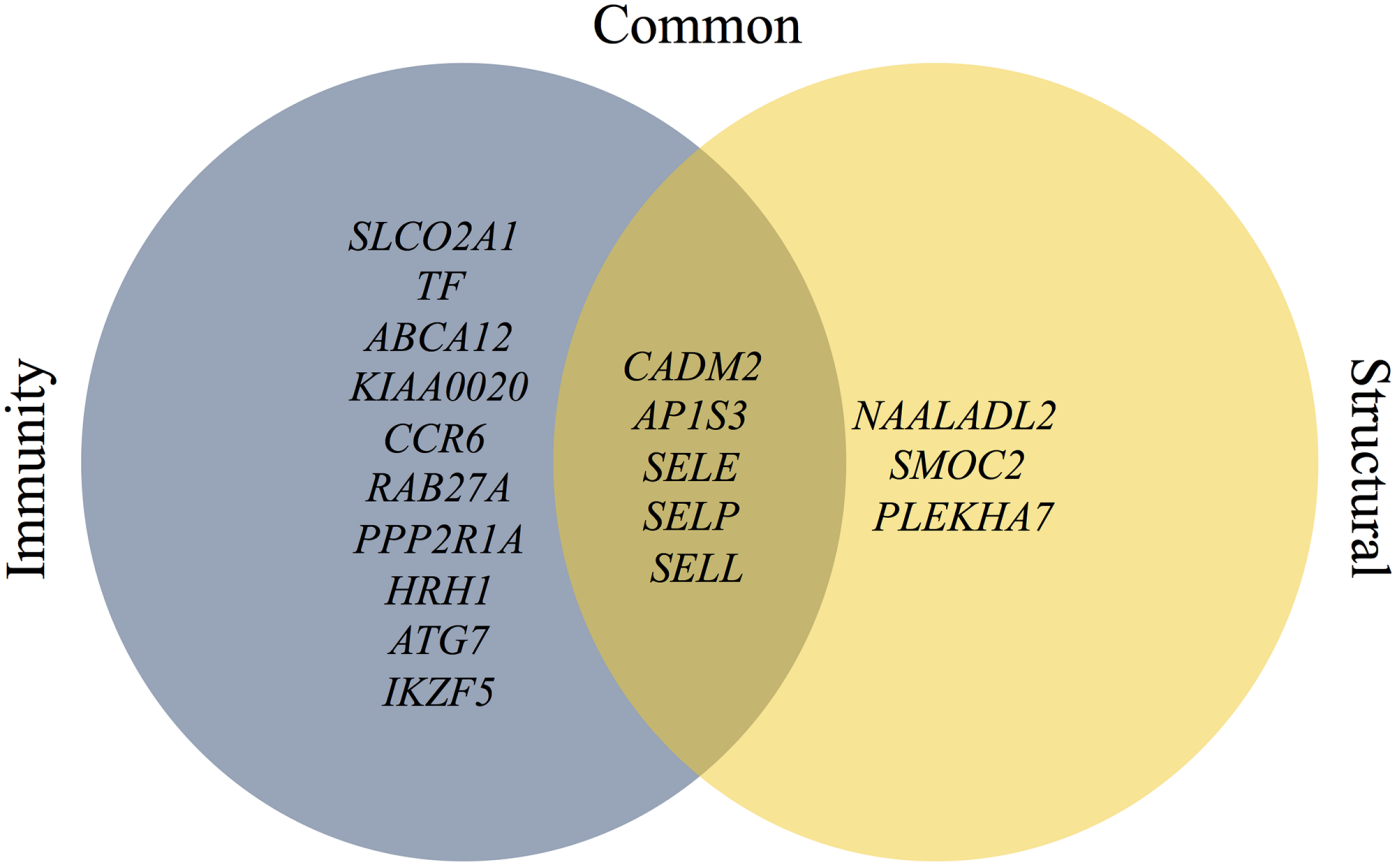
Genes and systems. Structural, genes involved in repair/wound healing processes without apparent immune function, including: adhesive activity, extracellular matrix (ECM) remodeling, epithelial-to-mesenchyme transition (EMT) and profibrotic activities; Immunity, genes involved in immunological activity; Common, genes involved in both.

Within the 15 candidates that possess various immune functions, 5 genes, *CADM2* [38] *AP1S3* [39], *SELE* [40,41], *SELP* [41] and *SELL* [5,41,42], are associated with adhesive activity, ECM remodeling, epithelial-to-mesenchyme transition (EMT) and profibrotic activities. All are part of the repair/wound healing process that might lead to the generation of adhesions (Fig 5). Ten genes, *SLCO2A1* [43], *TF* [44], *ABCA12* [45], *KIAA0020* [46], *CCR6* [47], *RAB27A* [48], *PPP2R1A* [49], *HRH1* (GeneCards, http://www.genecards.org/), *ATG7* [50,51] and *IKZF5* [52] are known for their immunological activity. Three genes, *NAALADL2* [53,54], *SMOC2* [55,56] (GeneCards), *PLEKHA7* [57,58], possess the above scar formation activities without apparent immune function (Fig 5). Thus, although they may respond to immunological cues [32], it is tempting to refer to these three candidates as potential exclusive markers for kosher status while the other 15 may serve as markers for both BRD and kosher status. Interestingly, Tizioto *et al*. [27] showed that one of these three genes, *SMOC2*, was differentially expressed in lymph nodes in response to BVD infection. It belongs to a protein family that mainly presents in tissues undergoing repair or remodeling [59]. Possibly, in response to BVD infection, *SMOC2* plays a role as an early wound repair gene.

The definition of the non-Kosher status as a phenotype characterized by pulmonary adhesions makes the eight structural and common genes, namely, structural and immune functions, interesting candidates. In line with the above, functional polymorphism in genes reported herein may alter the molecular pathways involved in the wound healing process, rendering individuals susceptible/resistant to the lung adhesion phenotype. If this would occur in the three structural genes, it might exclusively affect the kosher phenotype. However, if it would take place also in the common and/or immunity genes, the pulmonary adhesion phenotype may be representative of BRD susceptibility/resistance as well.

A total of 2,253 AA substitutions were found in the NCBI dbSNP in 18 of the QTLR genes (Table 4). This polymorphism constitutes a potential source of structural changes of the proteins, and hence potentially a large number of quantitative alleles. It is more than enough to be a source of protein conformation alleles that differ from one another in their molecular efficiency. Thus, all QTLRs found in the present study harbor a plethora of candidate causative mutations potentially responsible for the quantitative effects found.

Such a polymorphism in protein coding genes is in accord with the AA polymorphism found in the chicken *OCX* gene by [60]. It suggests that a substantial portion of the AA may not be important to the protein function.

Another evidence that a gene may be a QTG is changing the level of expression in response to intrinsic and extrinsic cues. Tizioto *et al*. [27] searched for Differentially Expressed (DE) genes in cattle bronchial lymph node in response to a separate challenge by each of 3 viral and 3 bacterial BRD pathogens. Forty-three of the DE genes were located in the QTLRs of the present study (Table 5). On average 2.9 genes in a QTLR responded to a challenge by one or more pathogens. Fifteen of the QTLRs included genes that changed expression in response to at least one pathogen. QTLRs 3, 4, 8 and 18, contained genes that among them responded to all pathogens. All pathogens changed expression of genes in at least 4 QTLRs, averaging 9.3 QTLRs. The 43 genes found by Tizioto *et al*. [27] located in the QTLR of the present study responded to an average of 2.5 pathogens (Table 6), and 32 of these genes (74.4%) responded to more than 2 pathogens (Table 5). The six pathogens changed expression of an average of 27.7 of our 43 genes (5 to 30; Table 6).

**Table 5:** Tizioto *et al*. [27] differentially expressed genes found in the QTLRs of the present study.

Gene	QTLR	BTA	BRSV^c^	BVDV^d^	IBR^e^	*M*. *bovis*^f^	*M*. *haemolytica*^g^	*P*. *multocida*^h^	Pathogens^a^
*TF*	3	1	1	1	1	1	1	1	6
*SLCO2A1*	3	1	1	1	1	1	1	1	6
*BARD1*	4	2	1	1	1	0	0	0	3
*FARSB*	5	2	1	0	0	0	0	0	1
*KCNE4*	5	2	1	0	1	0	0	0	2
*VLDLR*	8	8	1	1	1	1	1	1	6
*RFX3*	8	8	1	1	1	0	0	0	3
*RNASET2*	9	9	1	1	1	0	0	0	3
*MLLT4*	9	9	1	0	1	0	0	0	2
*RAB27A*	10	10	1	1	1	0	1	0	4
*OTOG*	13	15	1	1	0	0	0	0	2
*SELP*	14	16	1	0	1	0	1	0	3
*SELL*	14	16	1	0	1	0	0	0	2
*BLZF1*	14	16	1	0	0	0	0	0	1
*LOC787057*	15	18	1	1	1	0	0	0	3
*ZNF614*	15	18	1	1	1	0	0	0	3
*LOC785630*	15	18	1	0	1	0	0	0	2
*ZNF432*	15	18	1	1	1	0	0	0	3
*VGLL4*	16	22	1	0	0	0	0	0	1
*DSC3*	17	24	1	1	1	0	0	0	3
*DMBT1*	18	26	1	1	1	0	0	1	4
*IKZF5*	18	26	1	1	1	0	0	0	3
*PLEKHA1*	18	26	1	1	1	0	0	0	3
*KIRREL3*	19	29	1	0	0	0	1	0	2
*EPHB1*	3	1	0	1	0	0	0	0	1
*AMOTL2*	3	1	0	1	0	0	0	0	1
*TOPBP1*	3	1	0	1	1	0	0	0	2
*FN1*	4	2	0	1	1	1	1	1	5
*SCG2*	6	2	0	1	1	0	1	0	3
*UNC93A*	9	9	0	1	1	0	0	0	2
*SMOC2*	9	9	0	1	0	0	0	0	1
*USH1C*	13	15	0	1	0	0	0	0	1
*NUCB2*	13	15	0	1	0	0	1	0	2
*SELE*	14	16	0	1	1	0	1	0	3
*SLC19A2*	14	16	0	1	1	0	0	0	2
*F5*	14	16	0	1	1	0	0	0	2
*HRH1*	16	22	0	1	0	0	0	1	2
*HTRA1*	18	26	0	1	1	1	1	1	5
*BFSP2*	3	1	0	0	1	0	0	0	1
*GRIA4*	12	15	0	0	1	0	0	0	1
*ZNF613*	15	18	0	0	1	0	1	0	2
*RAB6B*	3	1	0	0	0	0	1	0	1
*PRRX1*	14	16	0	0	0	0	1	0	1
		Genes^b^	24	29	30	5	14	7	109

^a^Number of pathogens affected the expression of the gene.

^b^Number of genes affected by the pathogen.

^c^Bovine respiratory syncytial virus.

^d^Bovine viral diarrhea virus Infectious.

^e^Bovine rhinotracheitis.

^f^Mycoplasma bovis.

^g^Mannheimia haemolytica.

^h^Pasteurella multocida.

**Table 6:** Effects of QTLR’s genes and pathogens.

	Pathogens/gene^a^	Genes/pathogen^b^		
		All	Virus	Bacteria
Avg	2.5	18.2	27.7	8.7
Min	1	5	24	5
Max	6	30	30	14

^a^Number of pathogens which changed the expression of a gene.

^b^Number of genes whose expression was changes by a pathogen.

Thus, most of the QTLRs found in this study based on the kosher phenotype, harbor an abundance of genes responding to BRD pathogens and thus are candidates for containing QTG.

Some of the QTLRs found in this study are in the vicinity of related QTLs mapped previously. QTL 2 on BTA 1 (Table 2) is within a QTLR affecting "Veterinary treatments" located at 91.9–97.3 Mb [61] and near a QTL located at 91.6–91.7 Mb affecting heat tolerance in beef cattle [62]. QTLR 5 on BTA 2 is near a BRD QTL [25]; QTLR 11 on BTA 12 and QTLR 18 on BTA 26 overlap BRD QTLs [14] and a BVD QTL region associated with the bovine viral diarrhea persistent infection, a virus frequently identified as a causative pathogen for BRD outbreaks [63]. QTL associated with carcass, production, reproduction and behavior traits were reported in different cattle breeds in the region of QRLR 19 on BTA 20 [64].

Being a part of the regular routine in kosher slaughterhouses, obtaining the kosher phenotype is free of charge, allowing large scale studies. The function and expression of tens of candidate genes, ampleness of candidate AA polymorphisms in the QTLR genes, and proximity of previously reported associated QTL, support the QTLRs found in this study, and thus support kosher phenotyping as an efficient means of mapping BRD QTL, QTG and causative mutations.

## Materials and Methods

### Samples and genotyping

All samples were collected as they came from male Holstein calves slaughtered in a commercial slaughterhouse (Adom Adom abattoir, Israel) under stringent kosher meat inspection requirements. Genetically, the Israel Holstein cattle population is very homogeneous. There is a single artificial insemination center that serves the entire country, so all female replacements are produced from the same pool of sires. Thus, the dams are a homogenous group and there is no reason to suspect population subdivision or stratification. The calves in the study population were produced over the course of a year, and hence represent a set of sire half-sib progeny groups. Examination of the pedigree of the study animals did not uncover any differential allocation of herds, sires, or maternal grandsires among the Glatt kosher and non-kosher groups. Hence, we believe that population structure was not a factor in our findings.

The inspector of the internal organs of the animal for assigning kosher status is trained to look for lung adhesions in the animal both before and after its lungs are removed. To test a lung, the inspector first removes all adhesions. If the lung is still intact it is classified as "Kosher"; Torn adhesions that cause perforations in the lung render it "Non- Kosher" (NK); Finally, lungs that are adhesion-free are referred to as “Glatt Kosher” (GK), referring to the fact that the animal’s lungs do not have any adhesions. In the current study GK individuals were taken as the High resistant group, while NK individuals were taken as the Low resistant group, as also recently appeared in Hayes *et al*. [65].

Blood was sampled immediately after slaughter, using evacuated tubes (Greiner Bio-One GmbH, Kremsmunster, Austria) containing EDTA as anticoagulant. DNA was isolated from the whole blood using Sigma DNA extraction kit, according to the manufacturer's instructions. DNA was quantified using NanoDrop (Wilmington, DE) spectrophotometry and purity was estimated using the 260/280 ratio. The quality control was performed on each sample to verify the DNA integrity on Invitrogen E-Gel 1% Agarose Gel.

Pools were constituted as reported by Strillacci *et al*. [66]. Five GK and two NK pools were made of 21 − 31 Holstein male calves each (Table 7).

**Table 7:** Number of individuals in the pools.

Pool^a^	Calves^b^
GK^c^	
1	27
2	28
3	23
4	21
5	23
NK^d^	
6	31
7	31
Total	
GK^c^	122
NK^d^	62
All	184

^a^Ordinal number of the pool.

^b^Number of calves in the pool.

^c^Glatt kosher.

^d^Non-kosher.

The pooled DNA samples were each genotyped in two duplicates, on two independent microarrays for a total of 14 microarray positions. Genotyping was performed at the University of Milan using the Illumina BovineHD BeadChip (777,962 SNPs). Each analysis was based on the genomic position of SNPs according to the bovine UMD3.1 genome assembly. Proportion in the pools of the Allele defined by Illumina as B (pB) was obtained by Illumina software.

#### Quality Control (QC)

Quality control filters were: SNP mapped to specific autosome location; no more than 50% or 25% missing genotypes for pools or markers, respectively; no more than a difference of 0.10 between the pB values of the two duplicates of the same pool-marker combination; and a minimum of 0.05 average marker pB. A total of 570,563 autosomal markers were retained after the QC and used to map QTLs.

### Statistical Methods

#### QTL Mapping: Testing for significance of marker-trait association

Let pB_ijk_ be the mean frequency of the B-allele across both duplicate arrays of the i^th^ marker in the j^th^ pool of the k^th^ tail.

i = 1 − M, where M is the total number of markers analyzed;

j = 1 − 5 for the GK pools, j = 1 − 2 for the NK pools;

k = 1 − 2, where 1 = GK pools, and 2 = NK pools.

We used a single-marker test for marker-trait association, where CWER P-value for the i^th^ marker was calculated as

P_i_ = 2 x the area of the standard normal curve to the right of

Zi=Di/SE(D) (1)

where,

D_i_ = pB_i.1_ − pB_i.2_;

pB_i.1_ = mean pB_ij1_ across the 5 GK pools;

pB_i.2_ = mean pB_ij2_ across the 2 NK pools.

For SE(D_i_) we used an empirical estimate of the standard erroof D_i_ under the null hypothesis of absence of marker-QTL association. This estimate is based on the variance among individual replicate pools within the same tail [67]. The basic assumption is that under the null hypothesis, the sampling variance among individual pools across tails is the same as the sampling variance among individual pools within tails. On this assumption, it follows from elementary principles, that

SE2(Di)=SE2(pBi.1)+SE2(pBi.2) (2)

=Var(pBi.1)/5+Var(pBi.2)/2 (3)

where,

Var(pB_i.1_) = the variance among the pB_ij1_ of the 5 replicate pools in the GK tail;

Var(pB_i.2_) = the variance among the pB_ij2_ of the 2 replicate pools in the NK tail.

We expect Var(pB_i.1_) = (VarpB_i.2_) to be the same, hence denoted Var(pB_i_). Then the best estimate of Var(pB_i_) will be the mean of Var(pB_i.1_) and Var(pB_i.2_) weighted by the number of degrees of freedom in each variance.

Var(pBi)=(4Var(pBi.1)+Var(pBi.2))/5 (4)

and

SE2(Di)=SE2(pBi.1)+SE2(pBi.2) (5)

=Var(pBi)/5+Var(pBi)/2=0.7Var(pBi) (6)

Because the estimate of Var(pB_i_) is based on a small number of pools, we used a global estimate of Var(pB_i_), averaged across markers and denoted Var(pB), as our estimate of the sampling variance of pB_i_ across pools. Because pB is a binomial variate, Var(pB) is a function of pB(1-pB), which maximizes at pB = 0.5 and drops off to either side. Thus, each marker requires an appropriate SE^2^(D), depending on its frequency. Consequently, we binned the markers in bins of width 0.1 from 0.0 to 1.0, according to their average pB across all pools (Avg pB_i_), and calculated average Var(pB) across the markers in each bin. Table 8 shows that indeed Var(pB) maximized in the range 0.4 to 0.6, dropping off to either side, thus justifying the use of Var(pB) according the marker mean pB. As a result, SE^2^D differed somewhat for the different markers, according to marker frequency in the pools.

**Table 8:** Weighted average of variances (Var(pB) of marker frequencies among replicates within GK and NK tails, pooled across all markers within bins.

Avg pB_i_	Var(pB)
≤ 0.1	0.00091
> 0.1–0.2	0.00562
> 0.2–0.3	0.00718
> 0.3–0.4	0.00796
> 0.4–0.5	0.00833
> 0.5–0.6	0.00847
> 0.6–0.7	0.00827
> 0.7–0.8	0.00716
> 0.8–0.9	0.00492
> 0.9–1.0	0.00109

Binning is by average marker frequency across all pools (Avg pBi).

The proportion of false positives (PFP; [37]) was used to correct for multiple tests.

#### QTL regions (QTLRs): Declaration of a QTLR

While searching for regions containing QTLs (QTLRs), significance of a single marker may not be enough to declare a QTLR, as a singleton significant P, without any support from surrounding markers, is prone to be false positive. On the other hand, visual inspection of chromosomal scatter charts reveals clusters of significant marker -LogP values (e.g., Fig 1). We interpret these clusters as putative QTLR. Note, however, that high -LogP values are interspersed with very low values across the cluster region. This behavior is related to complex LD patterns observed across small chromosomal regions [68]. Nevertheless, average -LogP value across a cluster region will be greater than in the adjoining regions lacking a concentration of high -LogP markers. Thus, to identify the clusters quantitatively, assign them an overall -LogP value, and determine their boundaries, we used a moving average of -LogP in 1 nucleotide steps taken across a window of markers. QTLRs were declared on the basis of windows having average -LogP above some chosen threshold. For the present study, average spacing between markers was 4,394 bp. Hence, windows of size 23 markers, equivalent to an average window size of 100 Kb were used. The size of a window was chosen to give a reasonable physical length yet not be overly influenced by the -LogP value of any single marker. A moving average of -LogP = 2, corresponding to P = 0.01, was set as the threshold value for declaration of a window as a QTLR. This threshold was set to give a reasonable number of ranked QTLR for further study in depth.

The windows worked well, and clear peaks with monotonic shoulders were obtained. See Fig 2 for a detailed example of the cluster of Fig 1. For this cluster, a run of 8 consecutive windows was found, all with a moving average above the chosen threshold of -LogP = 2. The top window among these had average -LogP = 2.470. The windows are located on the chromosome by the average location of their markers. The locations of the markers in the top window averaged 30,788,390, and this was taken as the point location of the QTL. The marker in this cluster with highest -LogP value (SNP BovineHD2900009163 located at 30,812,830 bp, -LogP = 7.304) was contained within this window, at a remove of 20,440 bp from the window average marker location.

#### Setting the boundaries of the QTLR

The popular LOD drop method [69] works well when applied to family-based linkage mapping and the low-density microsatellite maps of the previous generation of QTL mapping. In such studies, there is usually a monotonic inverse relation between marker significance and marker location relative to the most significant marker; with marker significance ascending monotonically marker by marker to the most significant marker and descending monotonically from that point. With GWAS and high-density marker maps, however, we are faced with the above-mentioned phenomenon where highly significant and non-significant markers are interspersed across the cluster. Consequently, there is no longer a monotonic relation between marker location and marker significance, making it impossible to apply the LOD drop method. That is, considering -LogP values of individual markers of the cluster, going out from the putative point location of the QTL (the most significant marker of the cluster), one will meet one or more non-significant markers, ostensibly setting a LOD drop boundary, but then just beyond these, are a new series of significant markers, clearly part of the same cluster and QTLR (e. g., Fig 2).

We found that the moving windows present a monotonic inverse relation of window location and window average -LogP values relative to the top window, forming a clear peak. This allowed the use of the LOD drop method for setting QTLR boundaries. For the present study, since we did not have LOD scores but -LogP, we used instead a -LogP drop of 1 (denoted "Log drop"). As noted above, for the cluster on BTA 29, the top window had average -LogP = 2.470. Accordingly, the Log drop 1 boundary of the QTLR were at -LogP = 1.470. The upstream and downstream windows with means closest to this value (-LogP = 1.470 and 1.492, respectively; Fig 2) were taken as the boundary windows. Since the windows are located by the average location of their markers, the actual boundaries of the QTLR were from the first marker of the upstream boundary window, to the final marker of the downstream boundary window. These markers were taken to define the final boundaries of the QTLR.

#### Distinguishing adjoining clusters

When two clusters and consequent runs of windows above the threshold were close to each other, two top windows and two peaks are seen (Figs 3 and 4). In this case, declaring the region as consisting of one or two QTLRs was based on the -LogP values of the region between the two top windows, relative to the lower of the Log drop 1 boundary thresholds of the two peaks. If the entire region between the peaks was above the higher Log drop 1 threshold, they were taken as one QTLR; otherwise, they were taken as two separate QTLRs. In the latter case, the exact boundary between the QTLRs was the window with lowest -LogP value.

### Bioinformatics

The SNPchiMp database [70] was used to convert the Illumina SNP name to the rsID (the SNP accession number used to search a SNP in all public databases). The rsID was used in NCBI database (http://www.ncbi.nlm.nih.gov/) to verify the precise position (intronic-exonic position, close to gene) of each SNP in respect to a gene. With one exception, none of the analyzed SNPs was in a coding region. SNP location and gene annotation were according to the UMD3.1.1 assembly. The full Ensembl v79 gene set for the autosomal chromosomes was downloaded (http://www.ensembl.org/biomart/martview/76d1cab099658c68bde77f7daf55117e). To identify the genes located within the QTLRs and inside 0.5 Mb intervals flanking the position of each SNP marker that define the same regions, we created a consensus list (among QTLRs and downloaded genes) using the BedTools software [71].

GO and pathway analyses were performed using the Database for Annotation, Visualization and Integrated Discovery (DAVID) v6.7 (http://david.abcc.ncifcrf.gov/). GO terms were used to categorize candidate genes in terms of their functions.

## Supporting Information

S1 Fig All autosomes. P values on all autosomes.
(PDF)

S1 Table Annotation of genes performed using DAVID on line database with high classification stringency option and the FDR correction. Sheet 1. SNPs report. Sheet 2. Genes clustered. Sheet 3: Genes not clustered. Sheet 4: Kegg pathways.
(XLSX)
