# Supplementary material for: The Use of Kosher Phenotyping for Mapping QTL Affecting Susceptibility to Bovine Respiratory Disease
Source: PLoS One. 2016 Apr 14;11(4):e0153423. doi: 10.1371/journal.pone.0153423 (PMC4831767; doi:10.1371/journal.pone.0153423)
Supplement: S1 Fig — P values on all autosomes. (PDF) [file pone.0153423.s001.pdf]

BTA 1

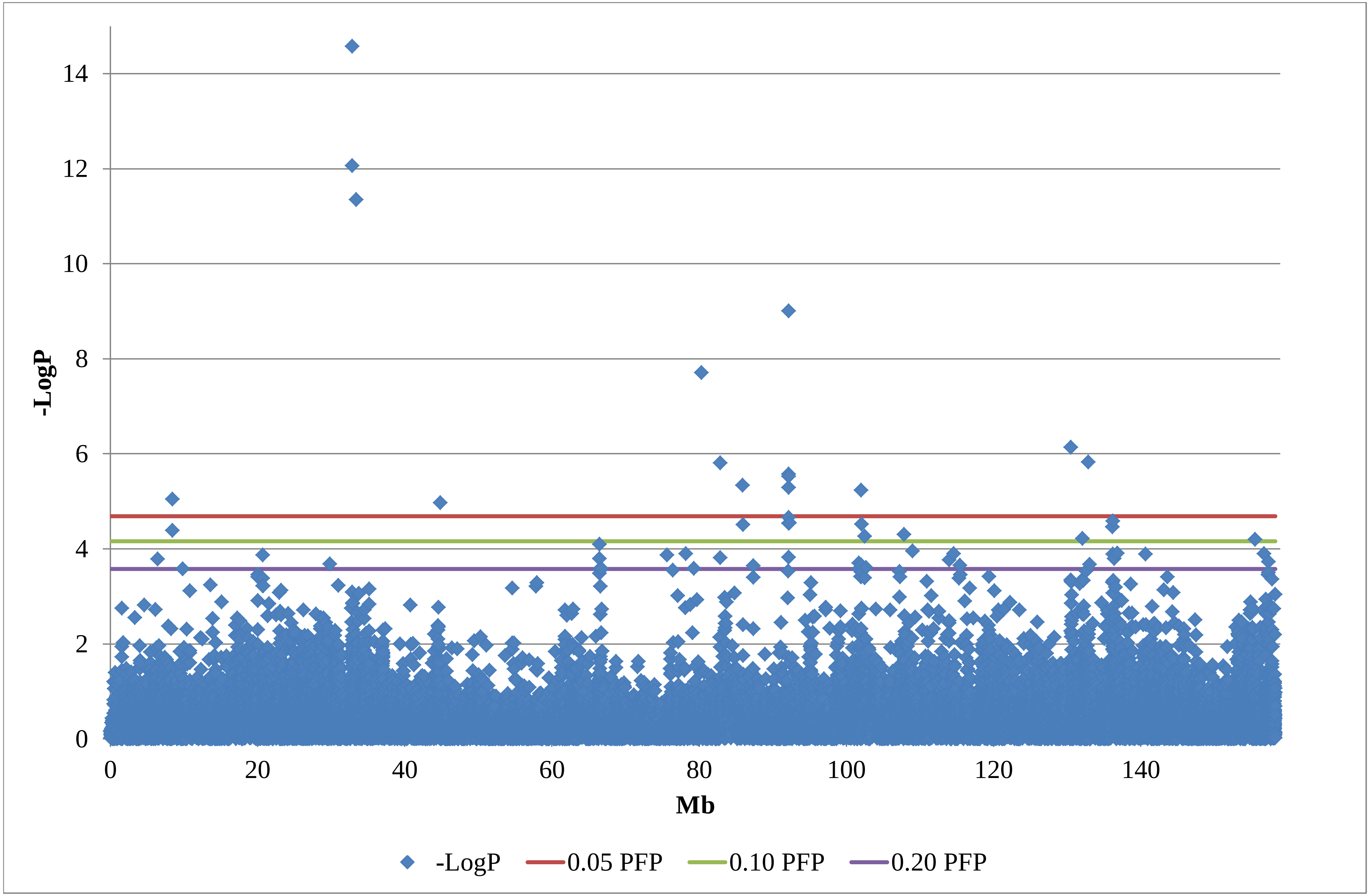

BRD pools -logP chr e.xlsx

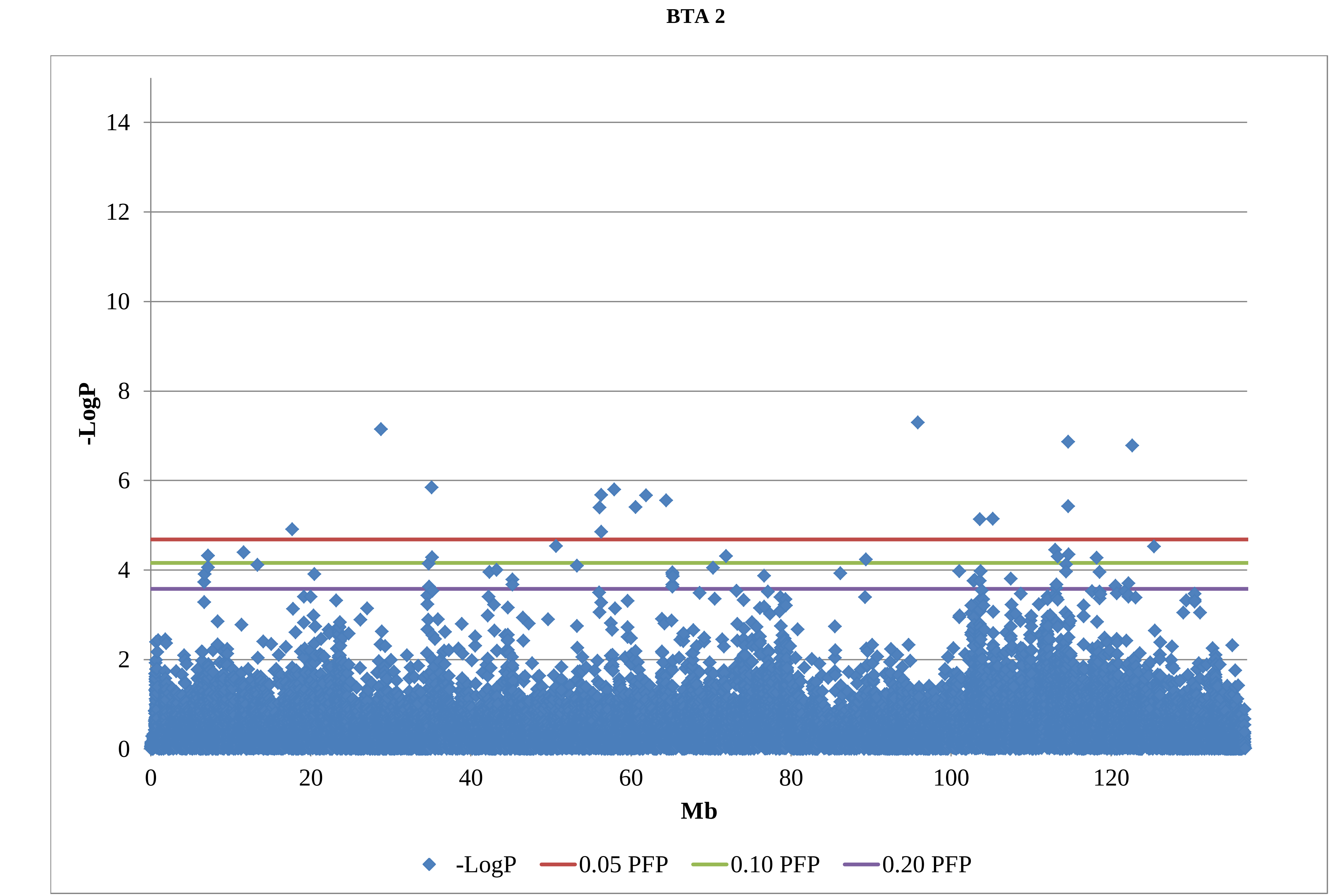
BTA 2
-LogP
14
12
10
8
6
4
2
0
0
20
40
60
80
100
120
Mb
-LogP
0.05 PFP
0.10 PFP
0.20 PFP

BTA 3

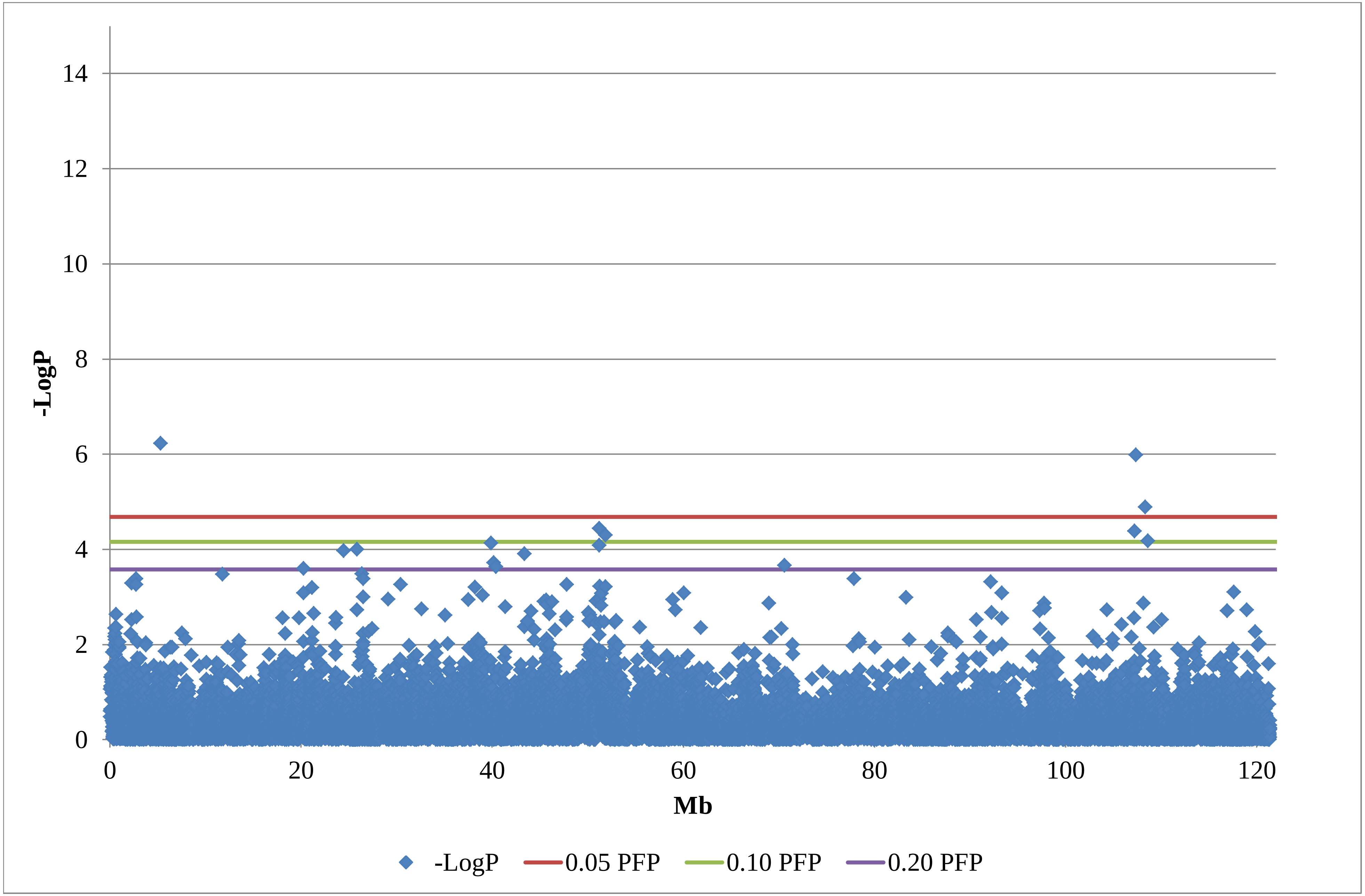
-LogP
0
2
4
6
8
10
12
14
0
20
40
60
80
100
120
Mb
-LogP
0.05 PFP
0.10 PFP
0.20 PFP

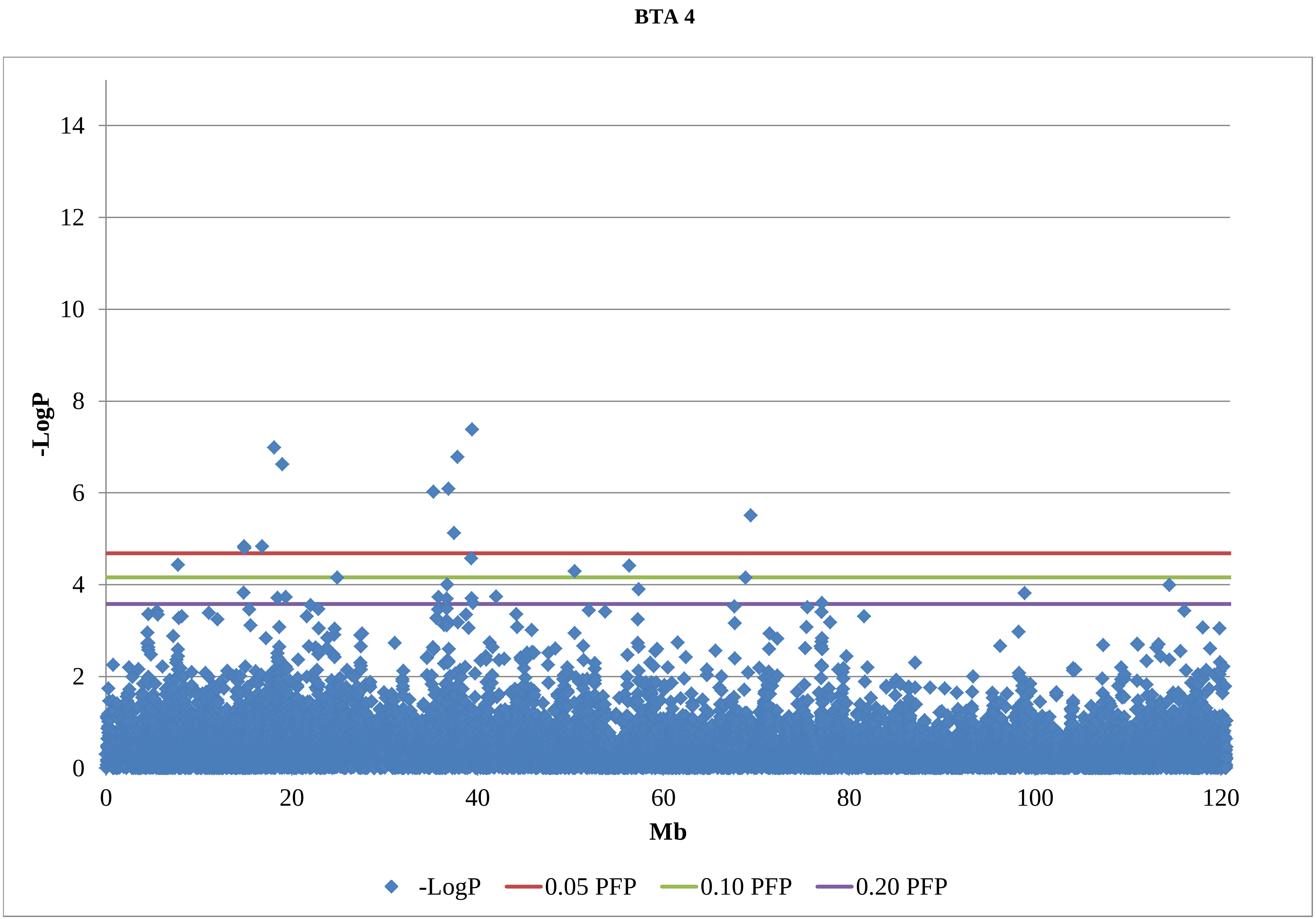

BRD pools -logP chr e.xlsx

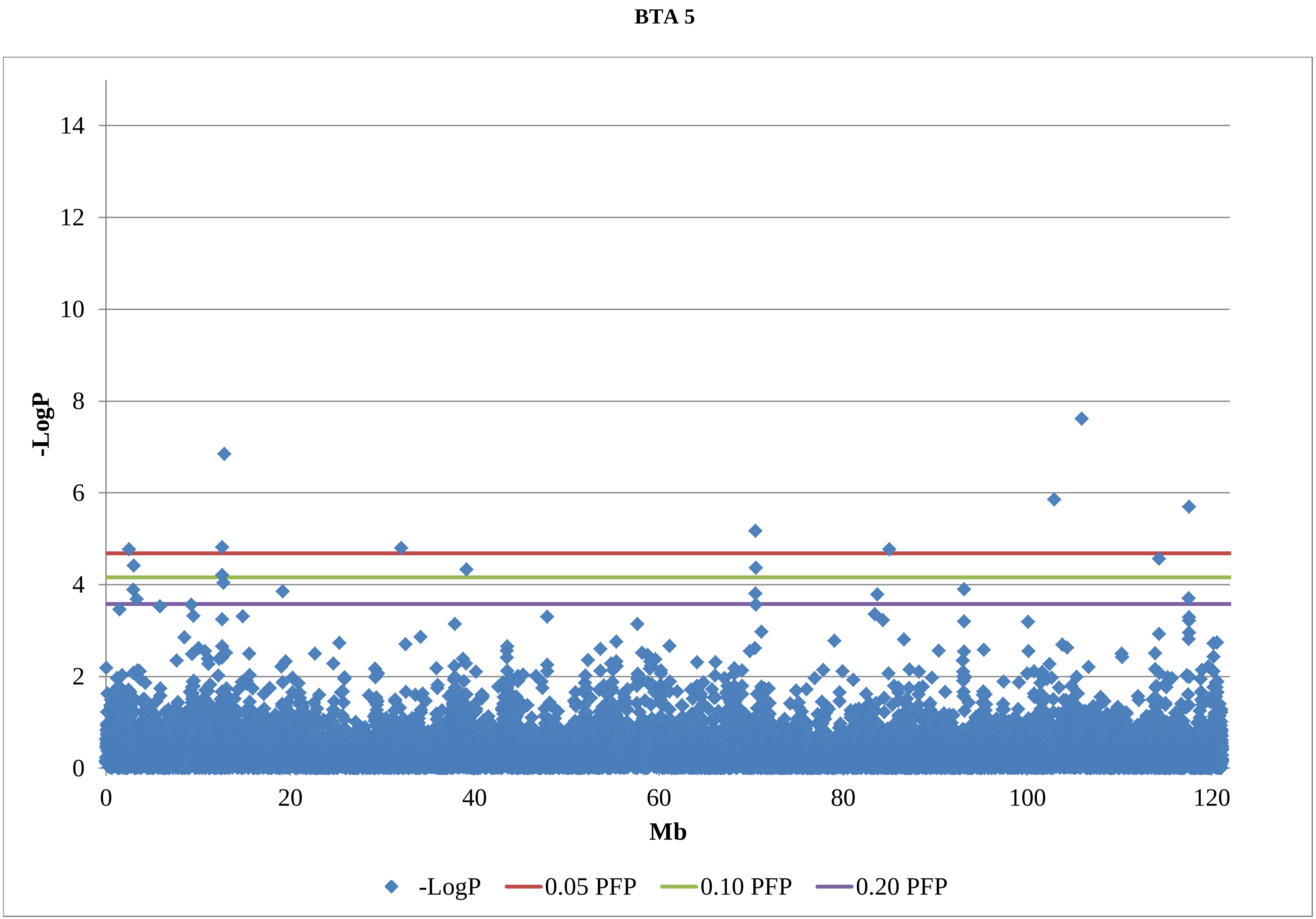
BTA 5
-LogP
14
12
10
8
6
4
2
0
0
20
40
60
80
100
120
Mb
-LogP
0.05 PFP
0.10 PFP
0.20 PFP

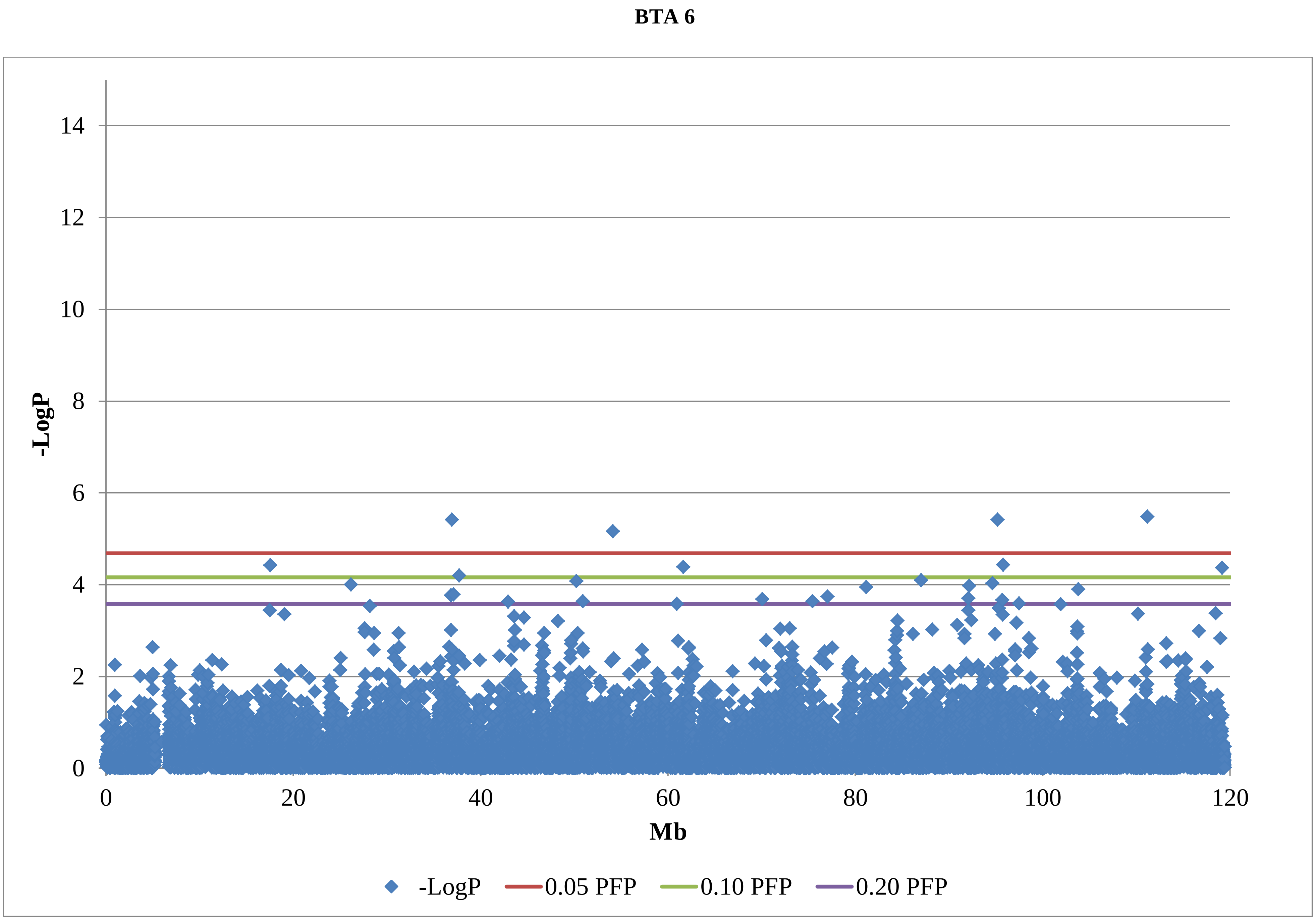

BRD pools -logP chr e.xlsx

BTA 7

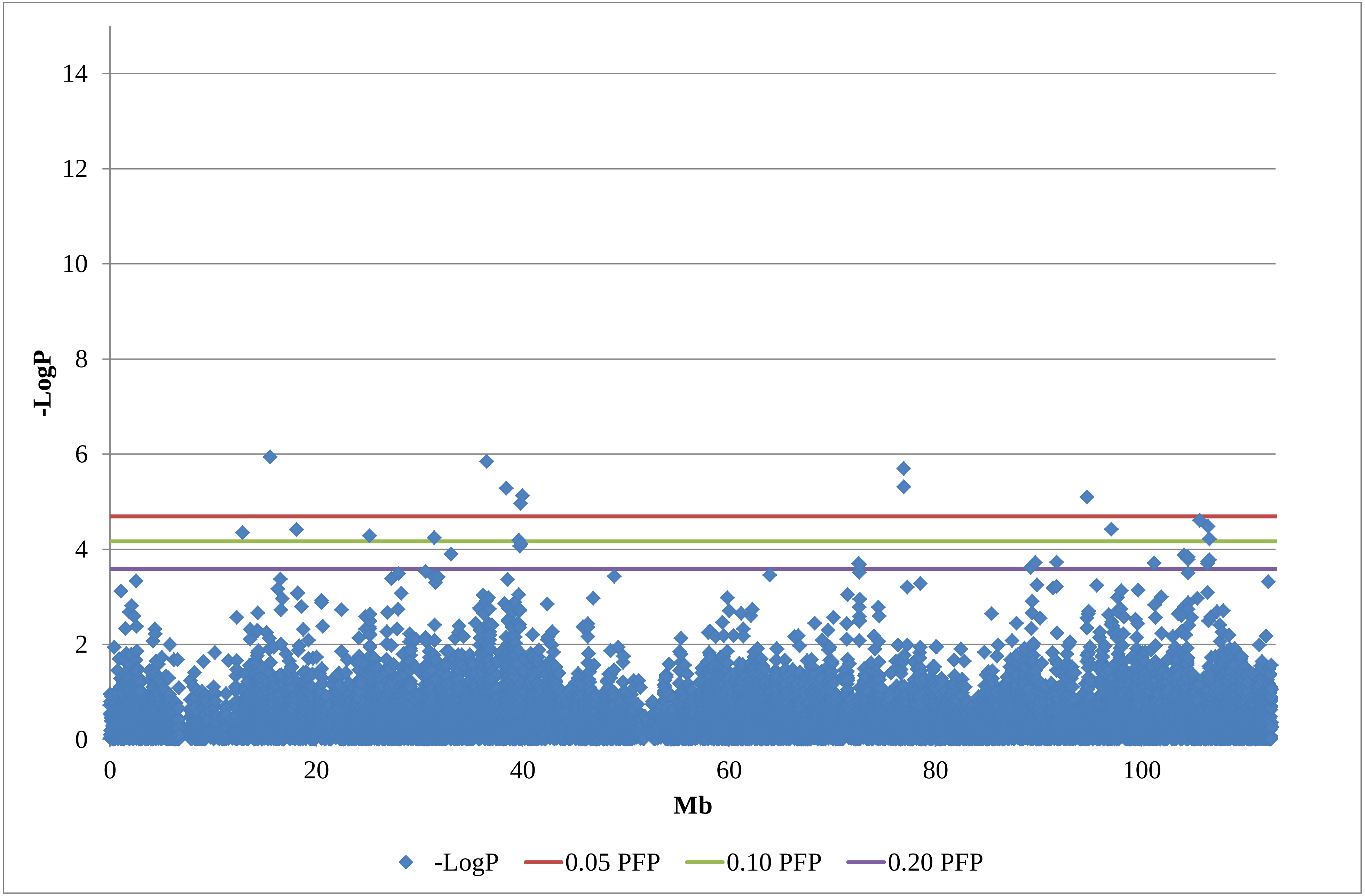

14
12
10
8
6
4
2
0
-LogP
0
20
40
60
80
100
Mb
-LogP
0.05 PFP
0.10 PFP
0.20 PFP

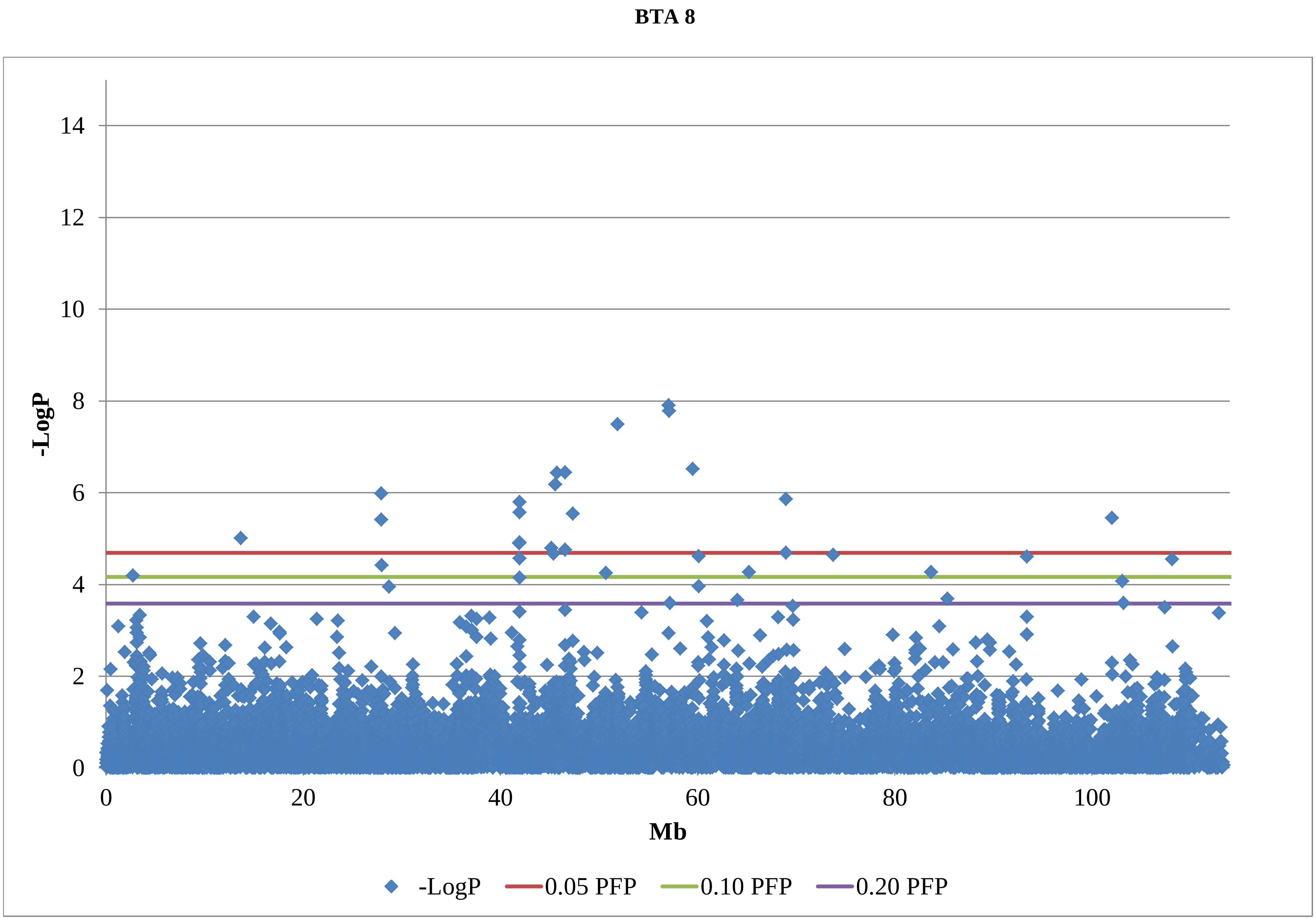

BRD pools -logP chr e.xlsx

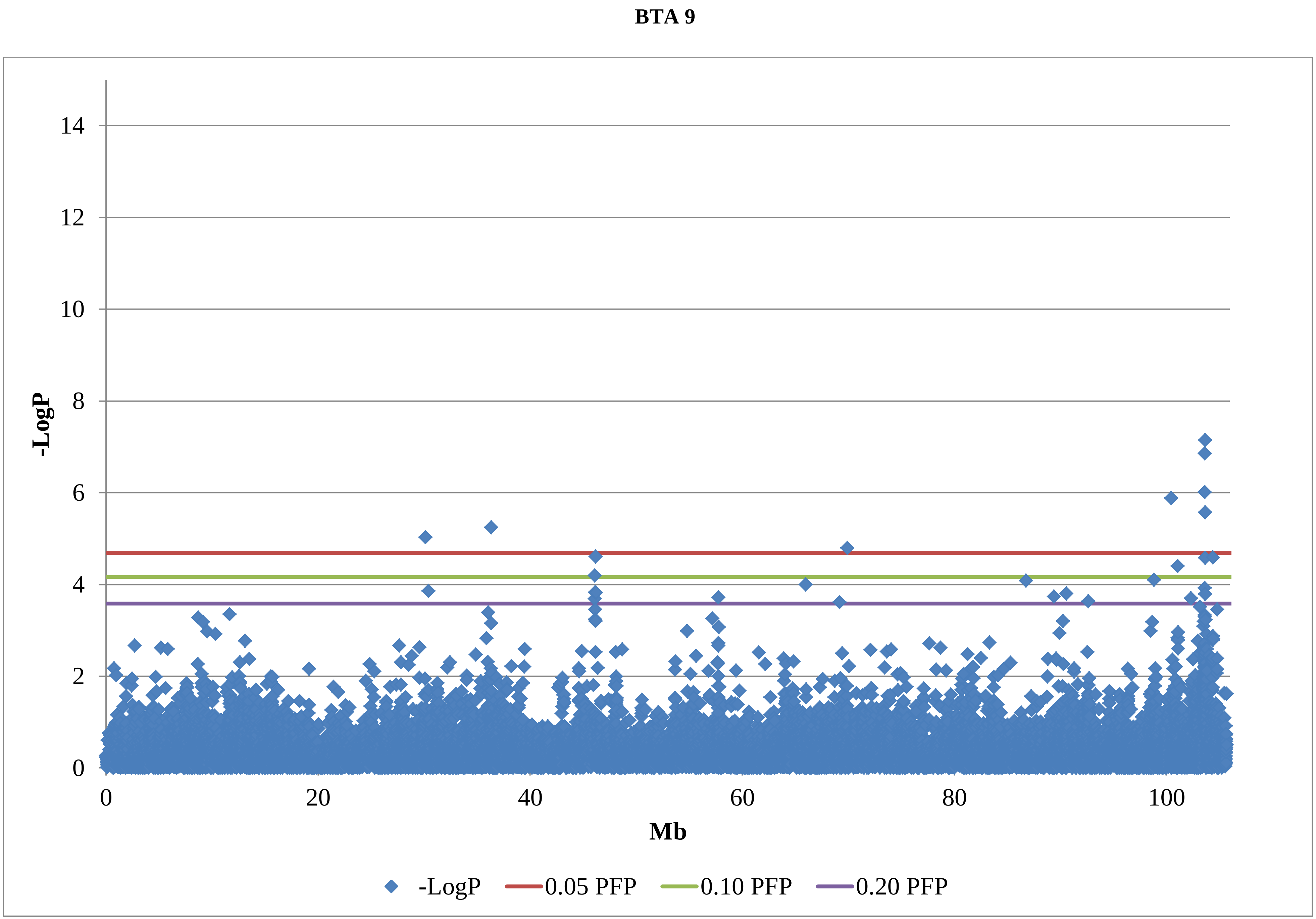

BTA 9
-LogP
14
12
10
8
6
4
2
0
0
20
40
60
80
100
Mb
-LogP
0.05 PFP
0.10 PFP
0.20 PFP

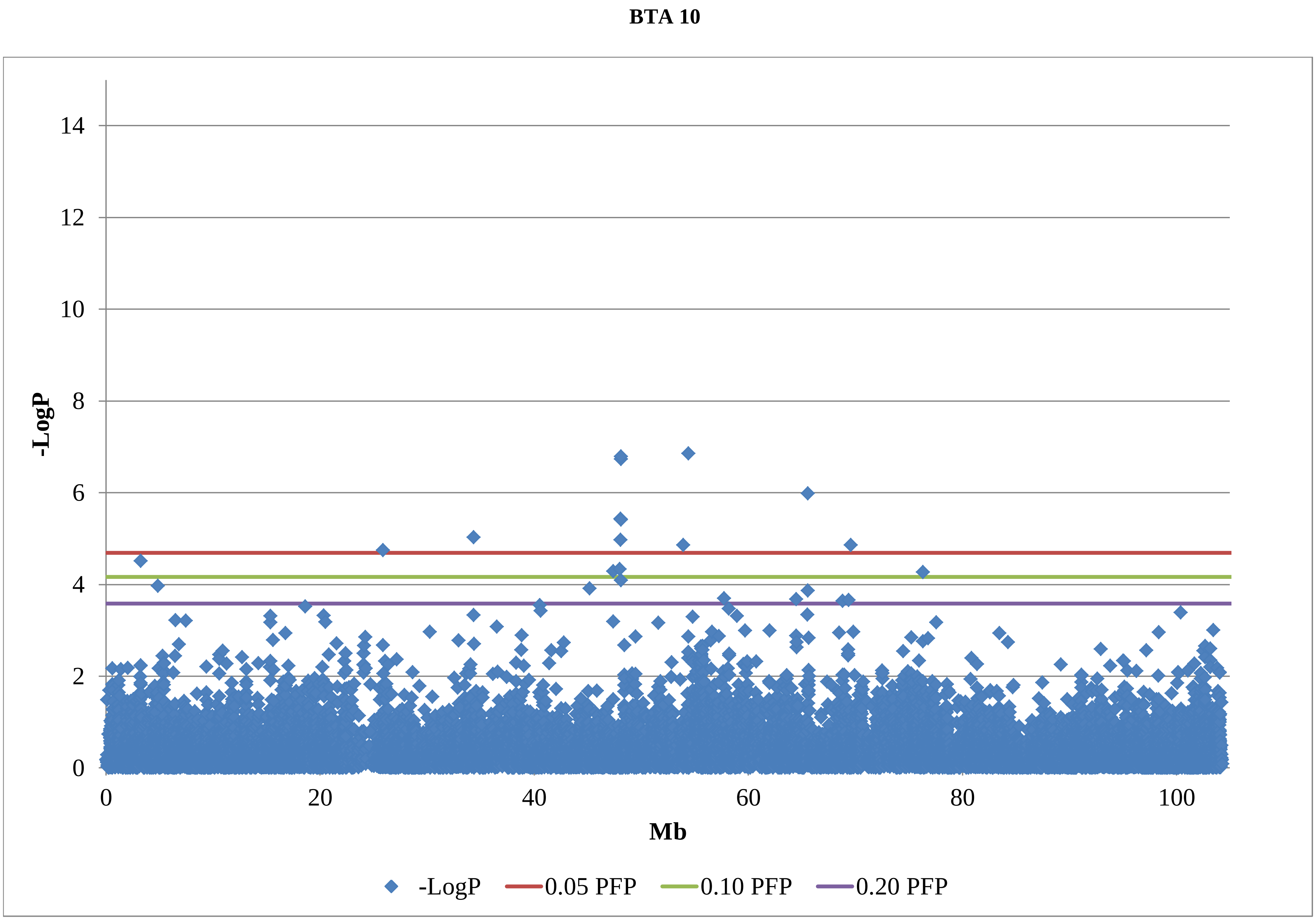

BTA 10
-LogP
14
12
10
8
6
4
2
0
0
20
40
60
80
100
Mb
-LogP
0.05 PFP
0.10 PFP
0.20 PFP

BTA 11

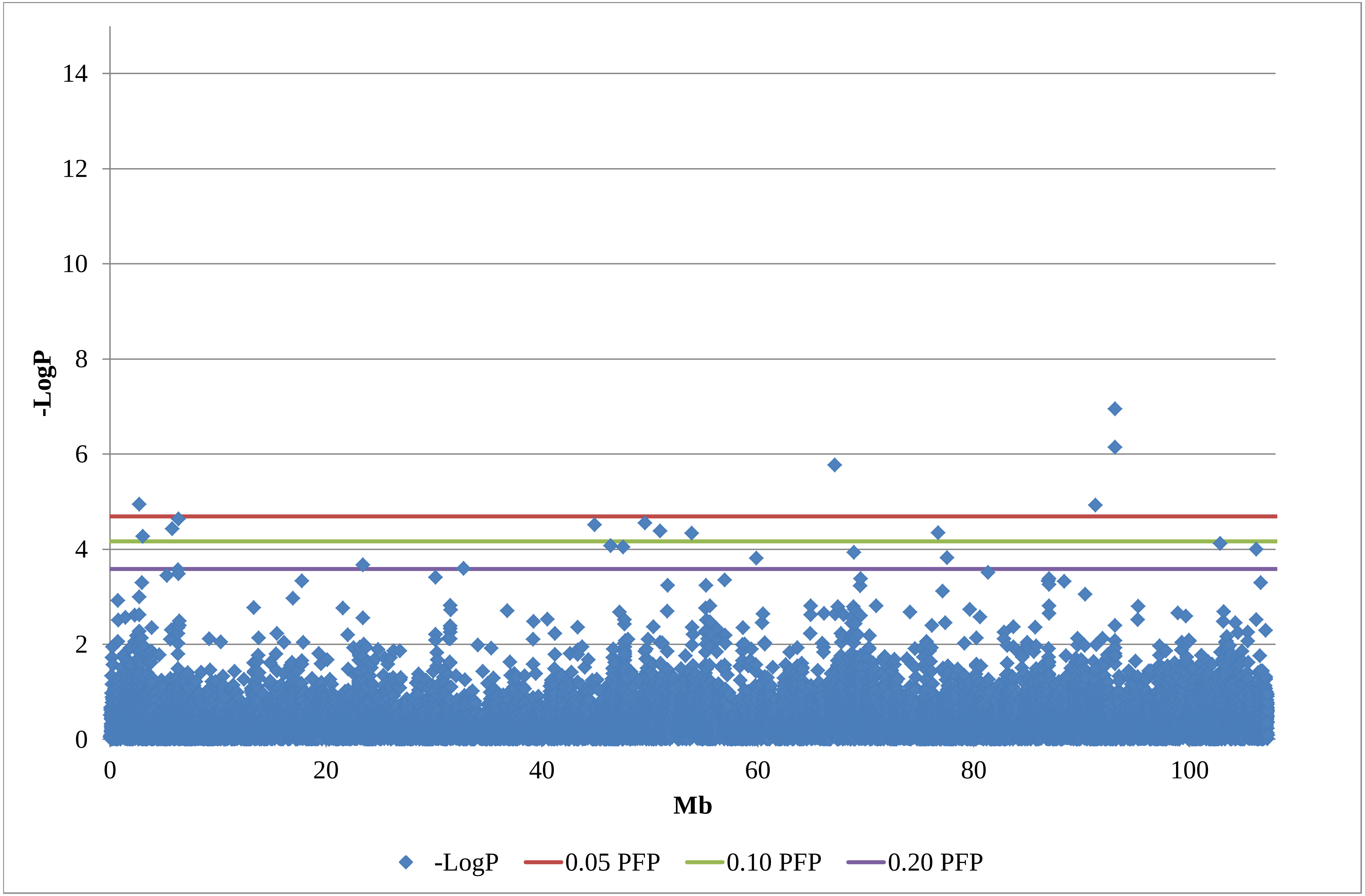

-LogP
14
12
10
8
6
4
2
0
0
20
40
60
80
100
Mb
-LogP
0.05 PFP
0.10 PFP
0.20 PFP

BTA 12

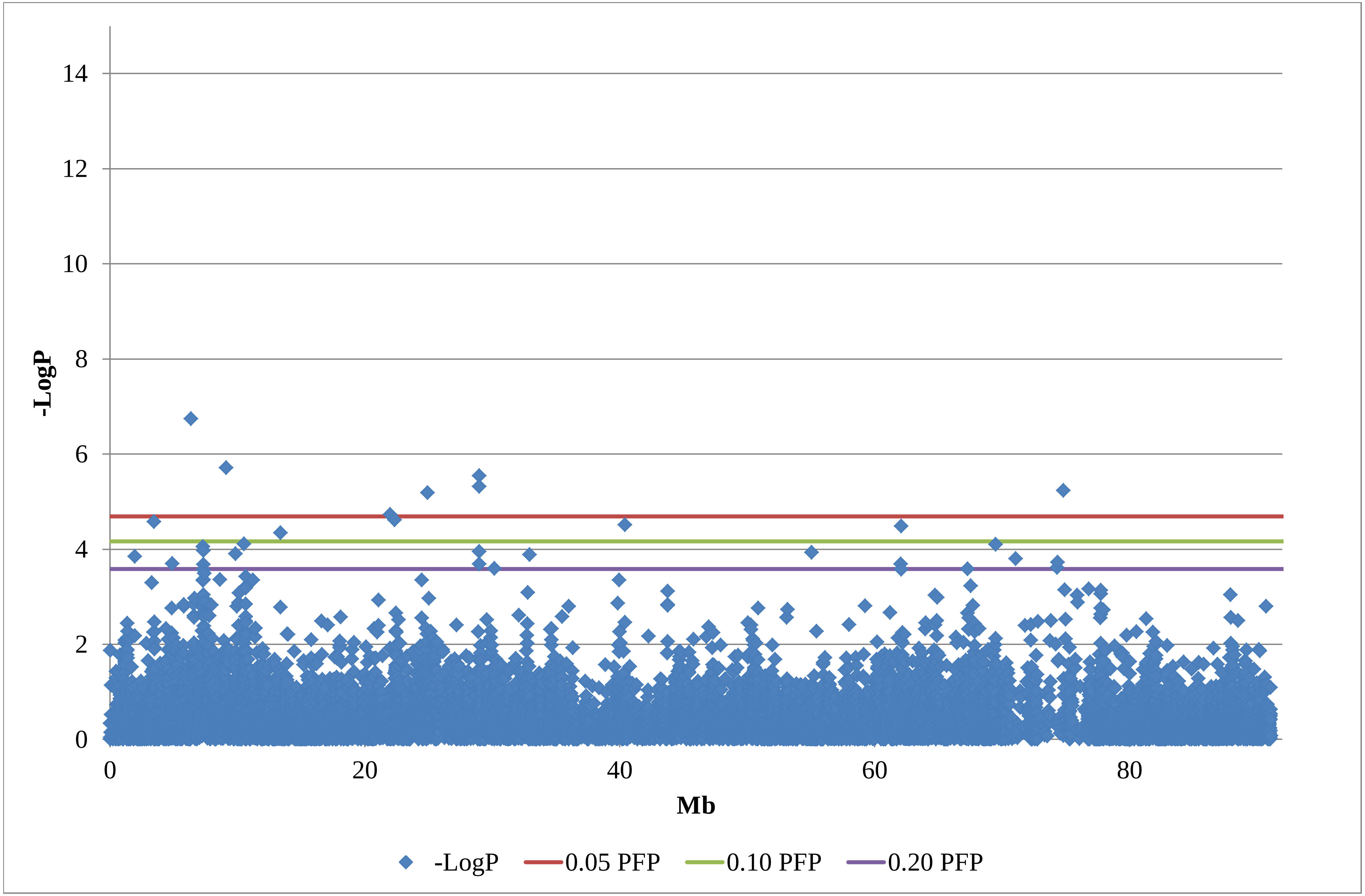

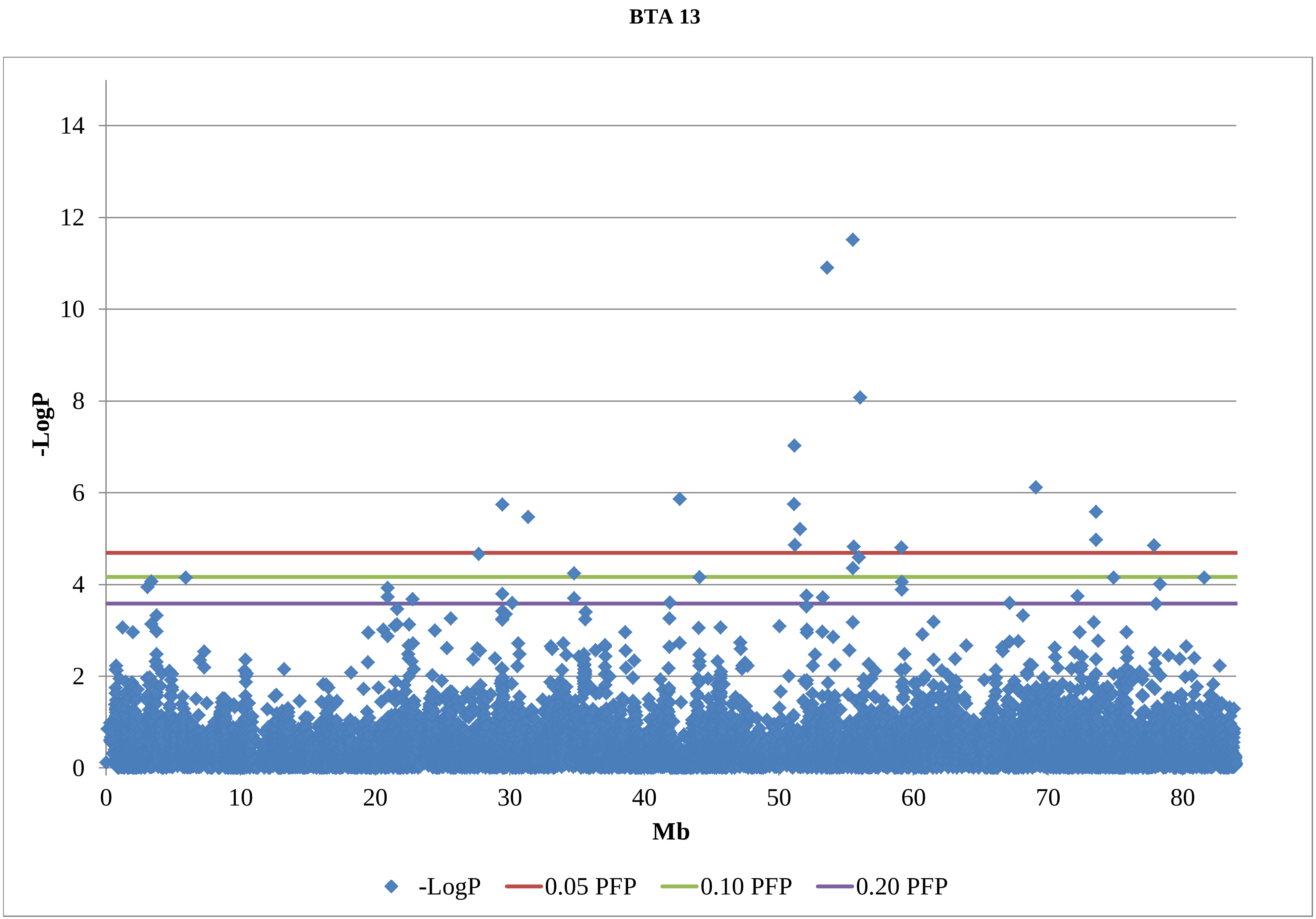

BRD pools -logP chr e.xlsx

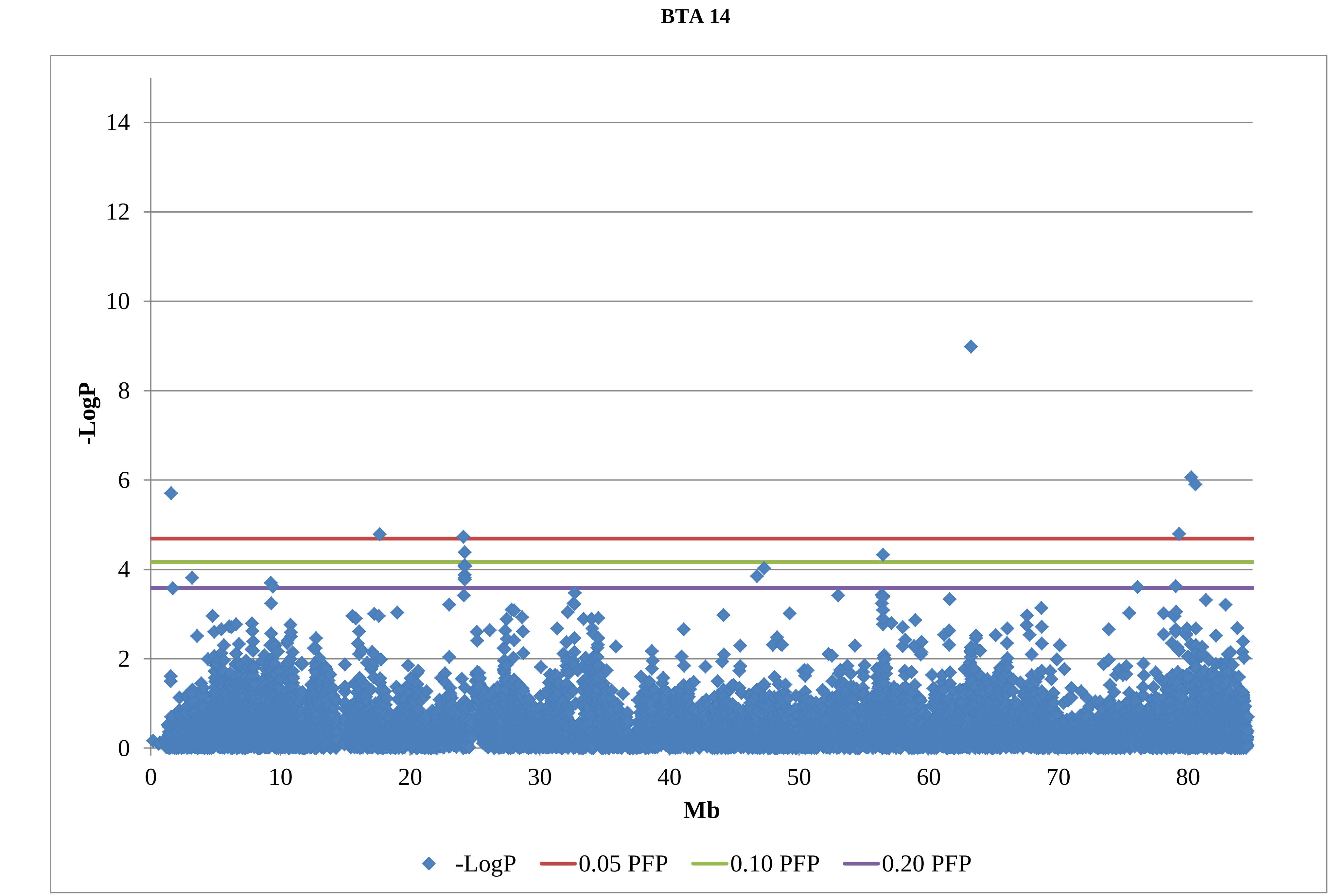

BRD pools -logP chr e.xlsx

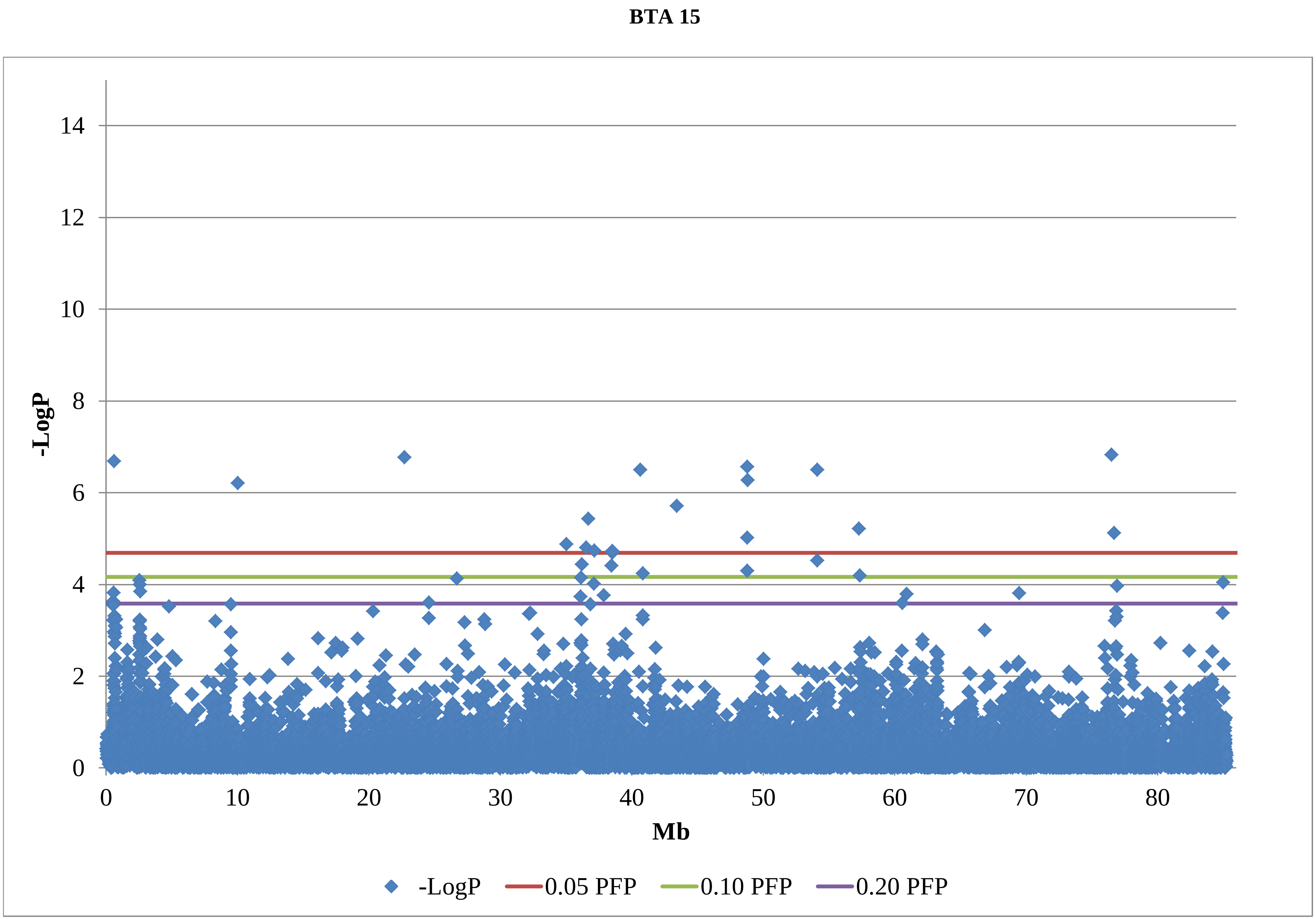

BRD pools -logP chr e.xlsx

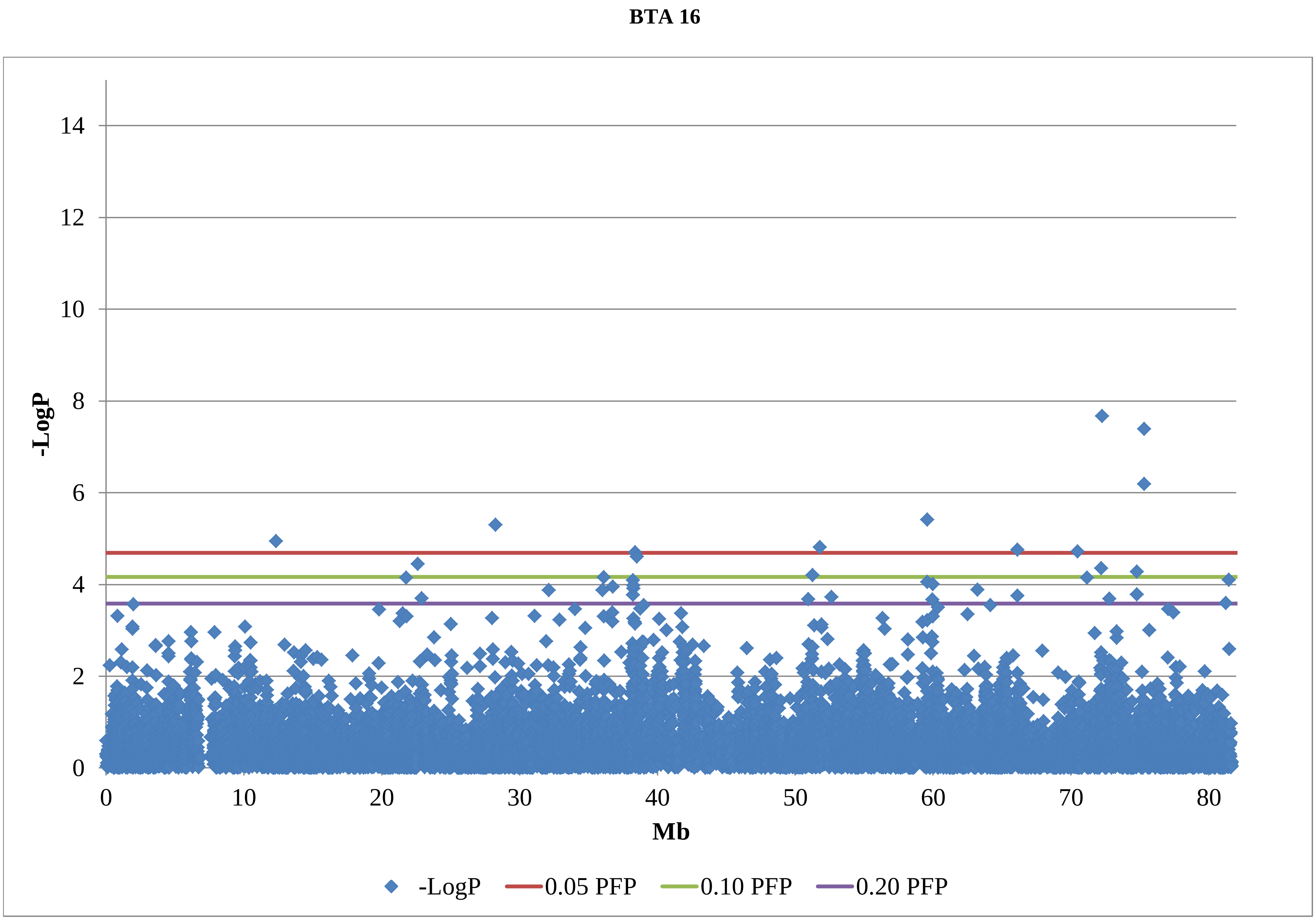

BRD pools -logP chr e.xlsx

BTA 17

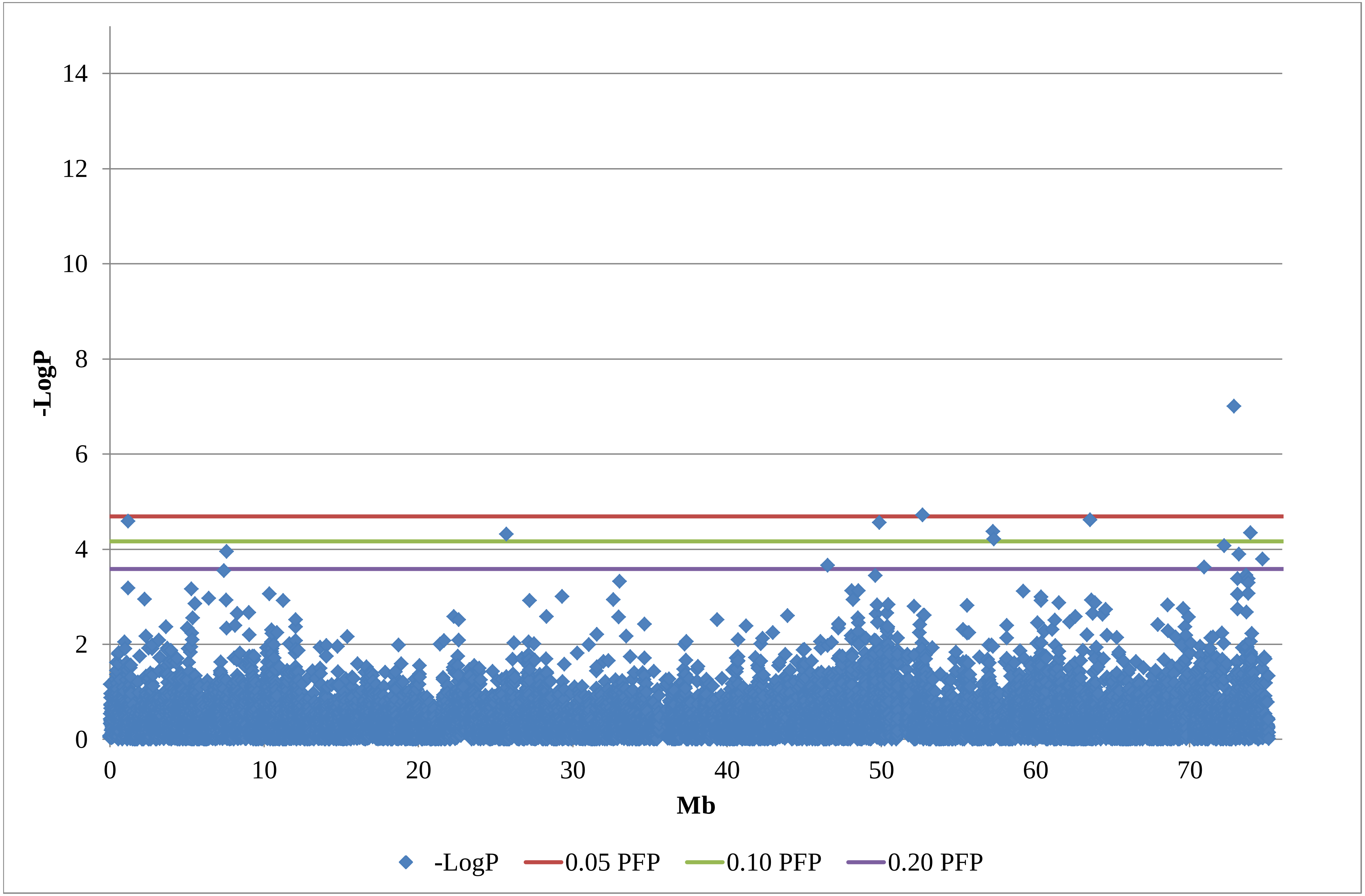

14
12
10
8
6
4
2
0
-LogP
0
10
20
30
40
50
60
70
Mb
-LogP
0.05 PFP
0.10 PFP
0.20 PFP

BTA 18

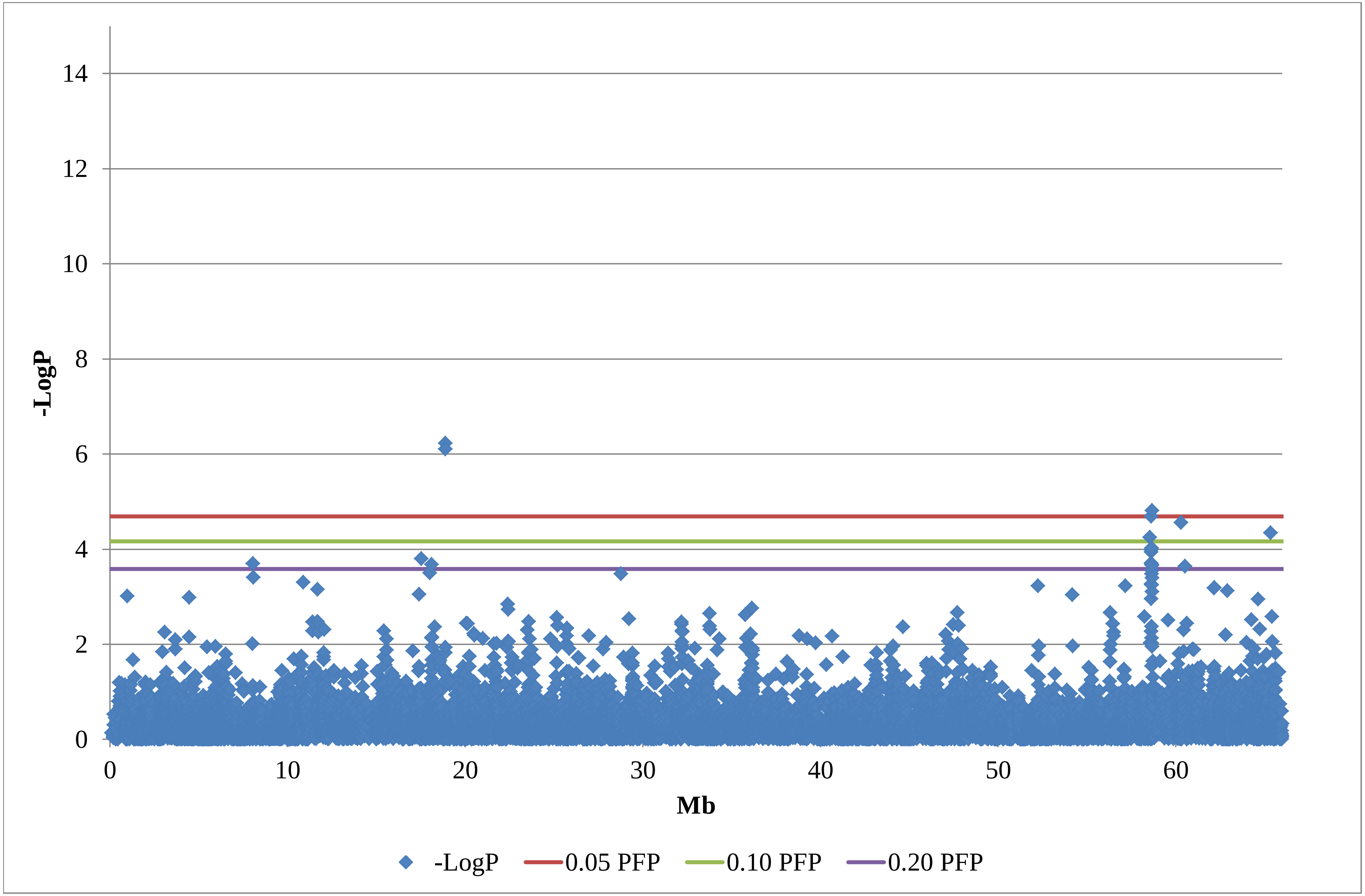

BRD pools -logP chr e.xlsx

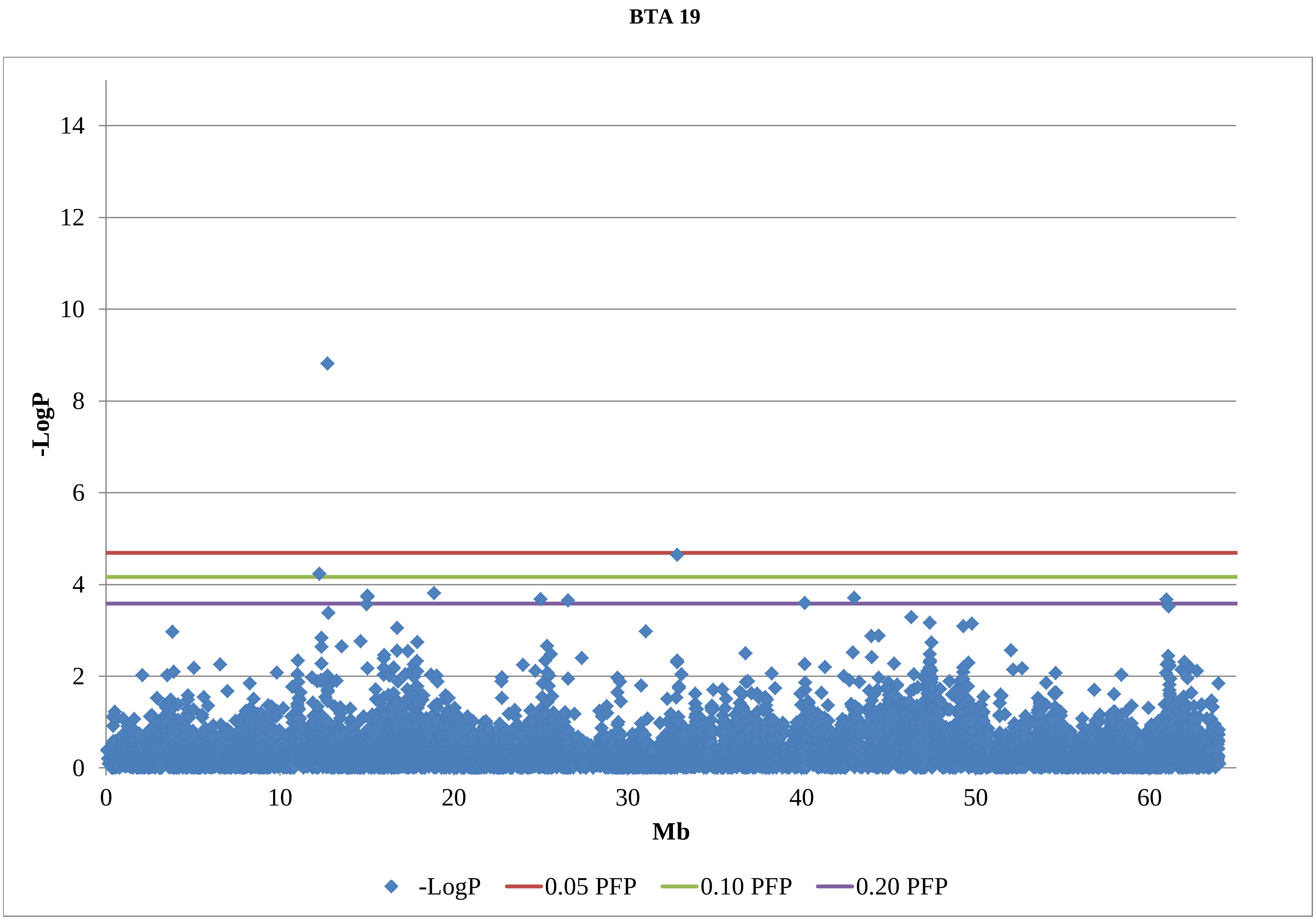

BTA 19
-LogP
14
12
10
8
6
4
2
0
0
10
20
30
40
50
60
Mb
-LogP
0.05 PFP
0.10 PFP
0.20 PFP

BTA 20

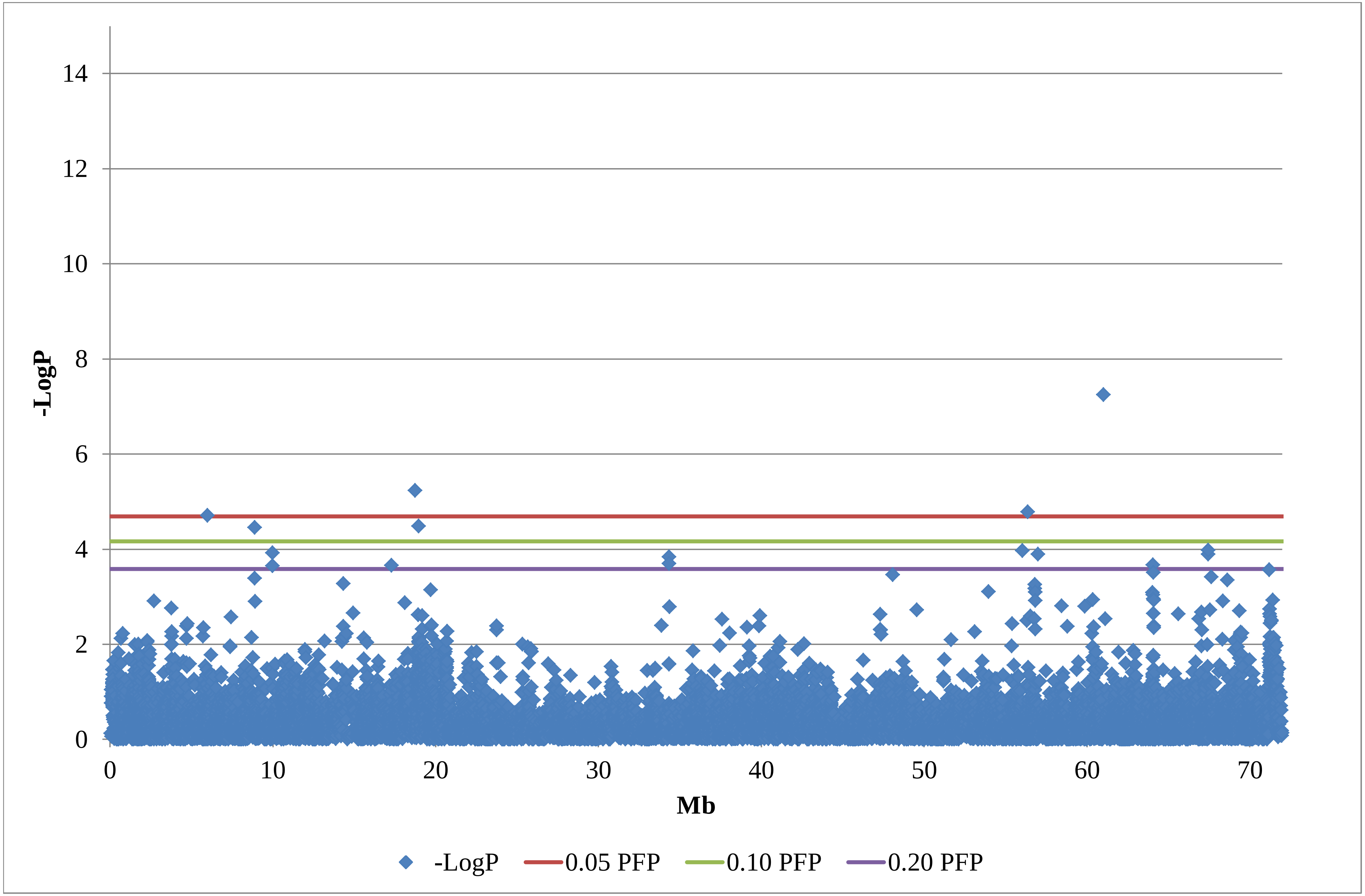

-LogP
14
12
10
8
6
4
2
0
0
10
20
30
40
50
60
70
Mb
-LogP
0.05 PFP
0.10 PFP
0.20 PFP

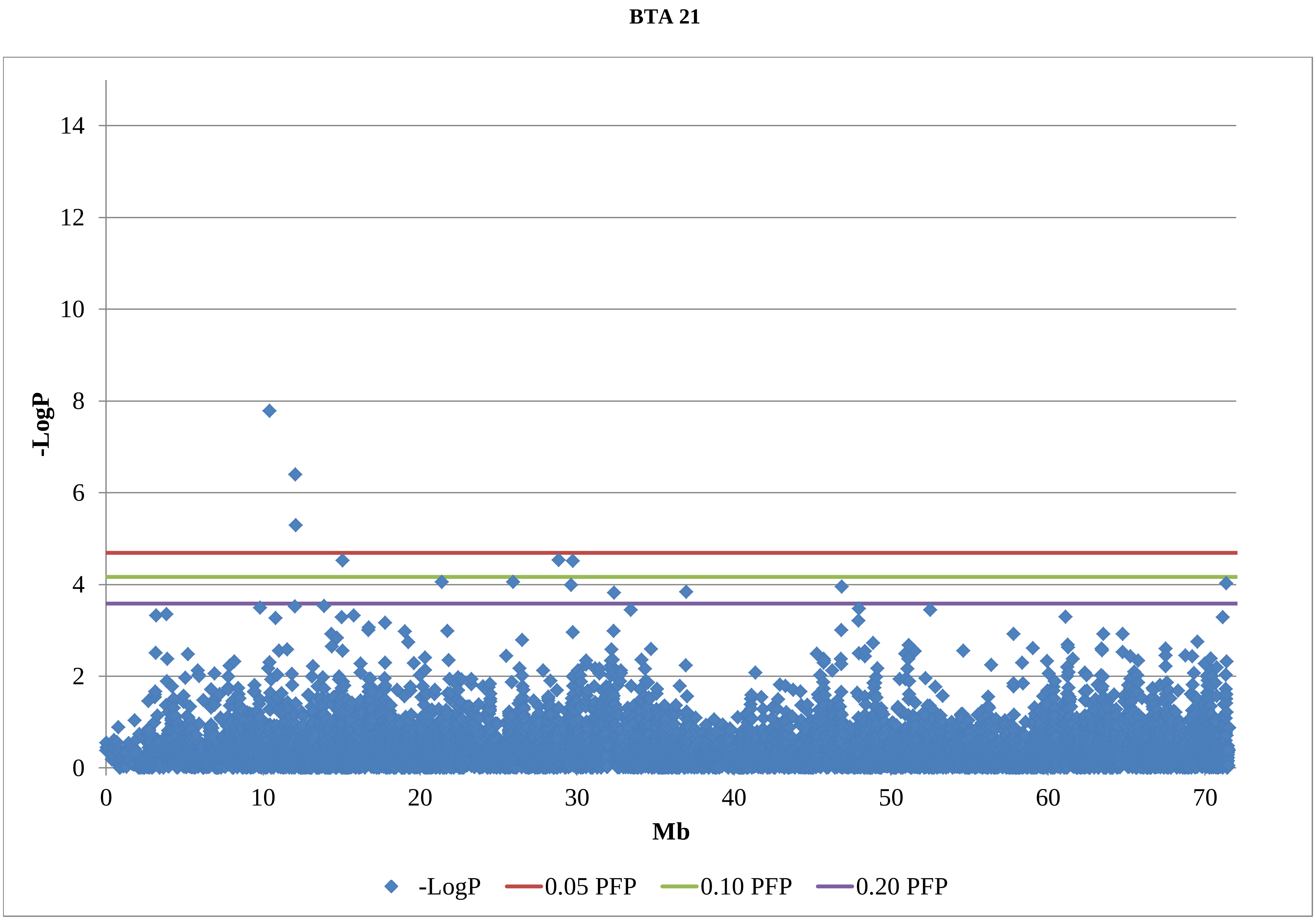

BRD pools -logP chr e.xlsx

BTA 22

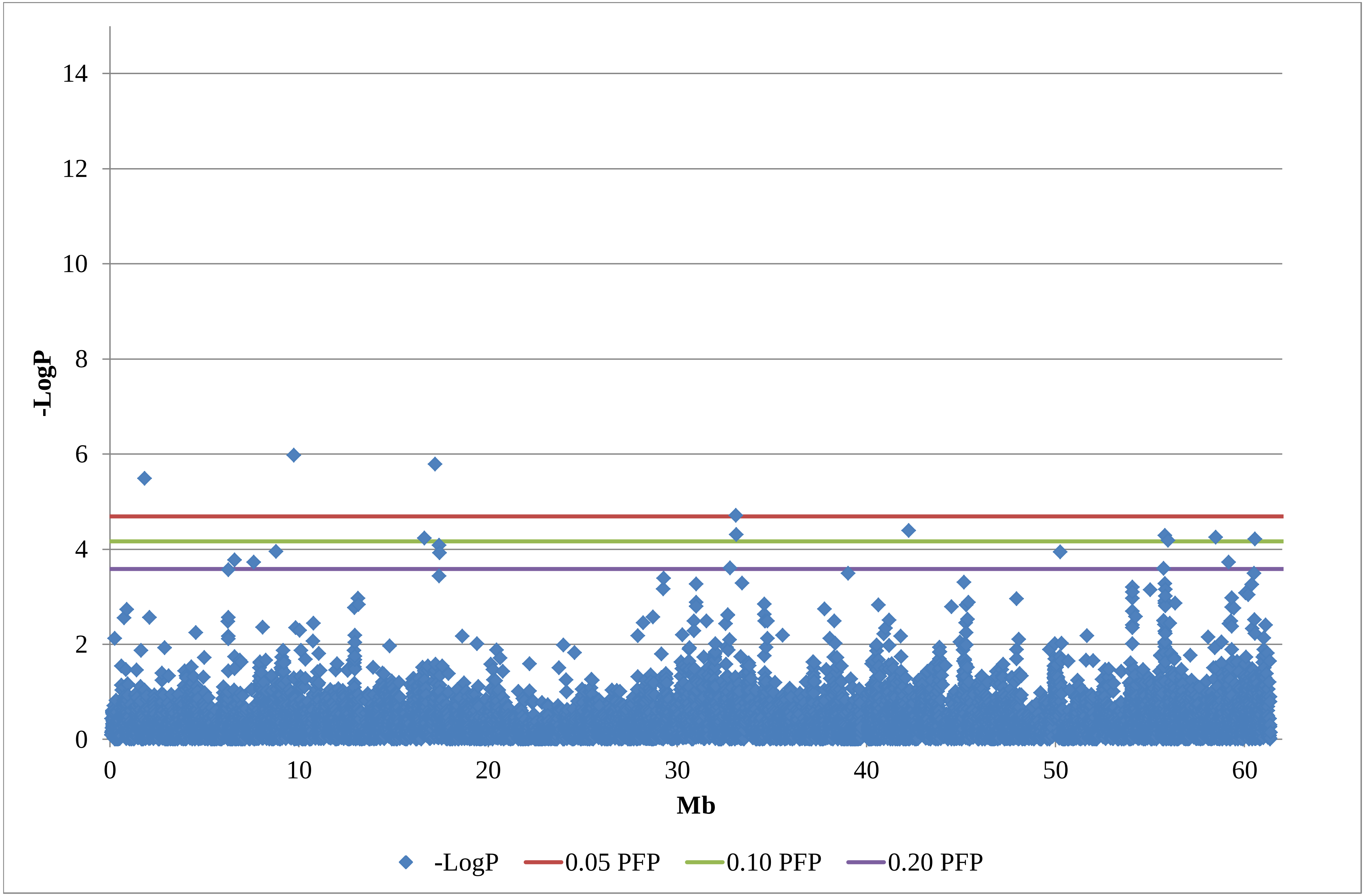

-LogP
14
12
10
8
6
4
2
0
0
10
20
30
40
50
60
Mb
-LogP
0.05 PFP
0.10 PFP
0.20 PFP

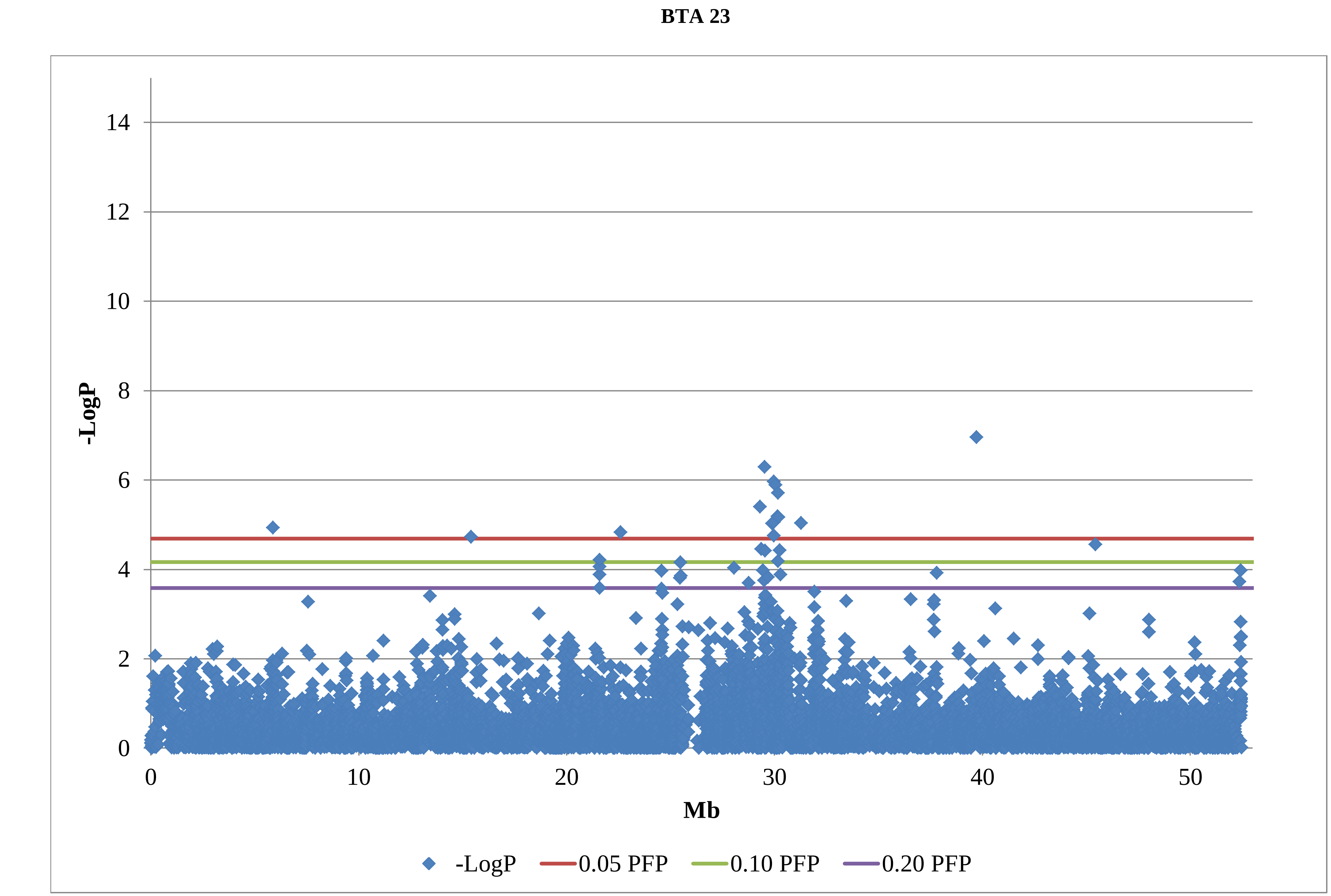

BRD pools -logP chr e.xlsx

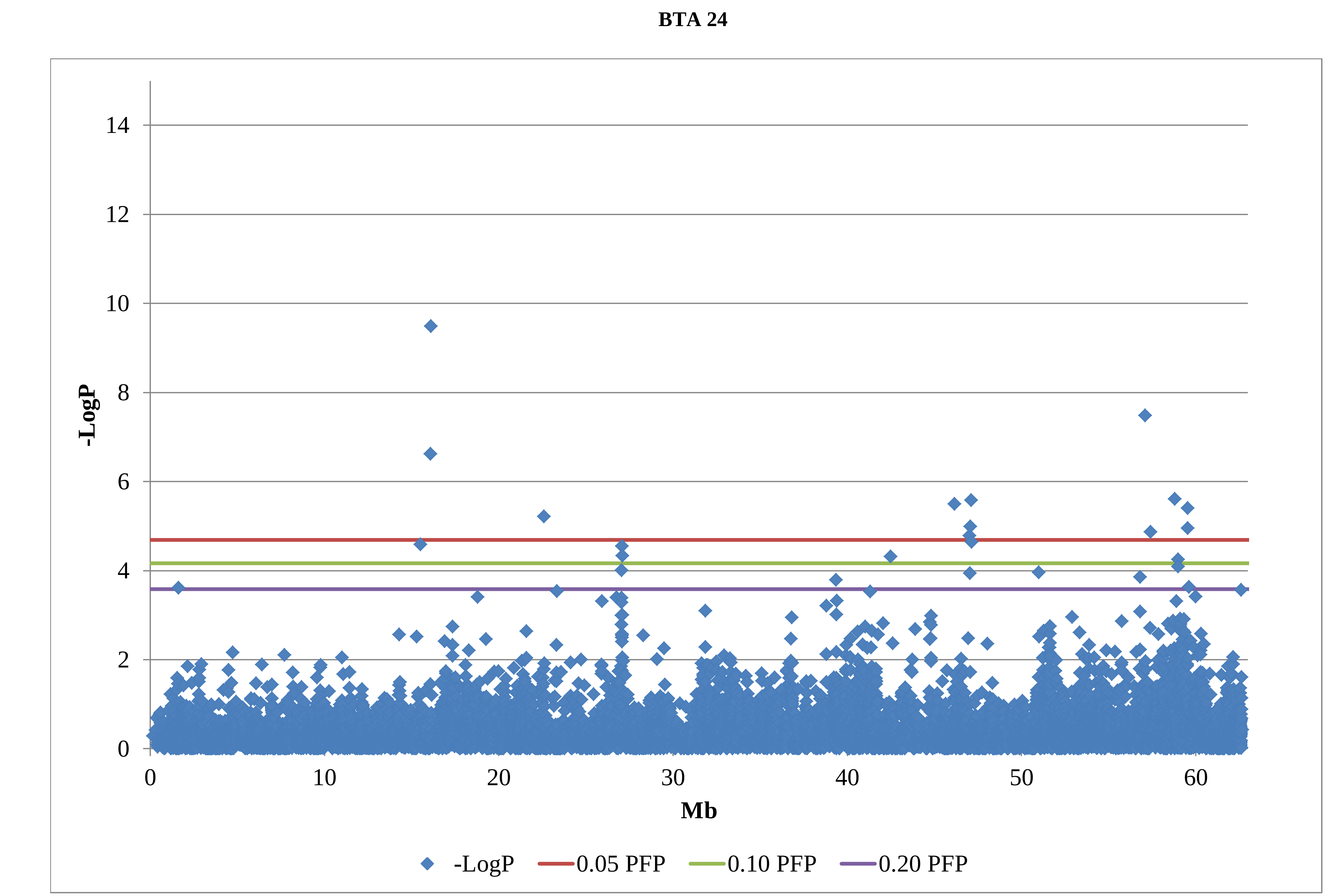

BRD pools -logP chr e.xlsx

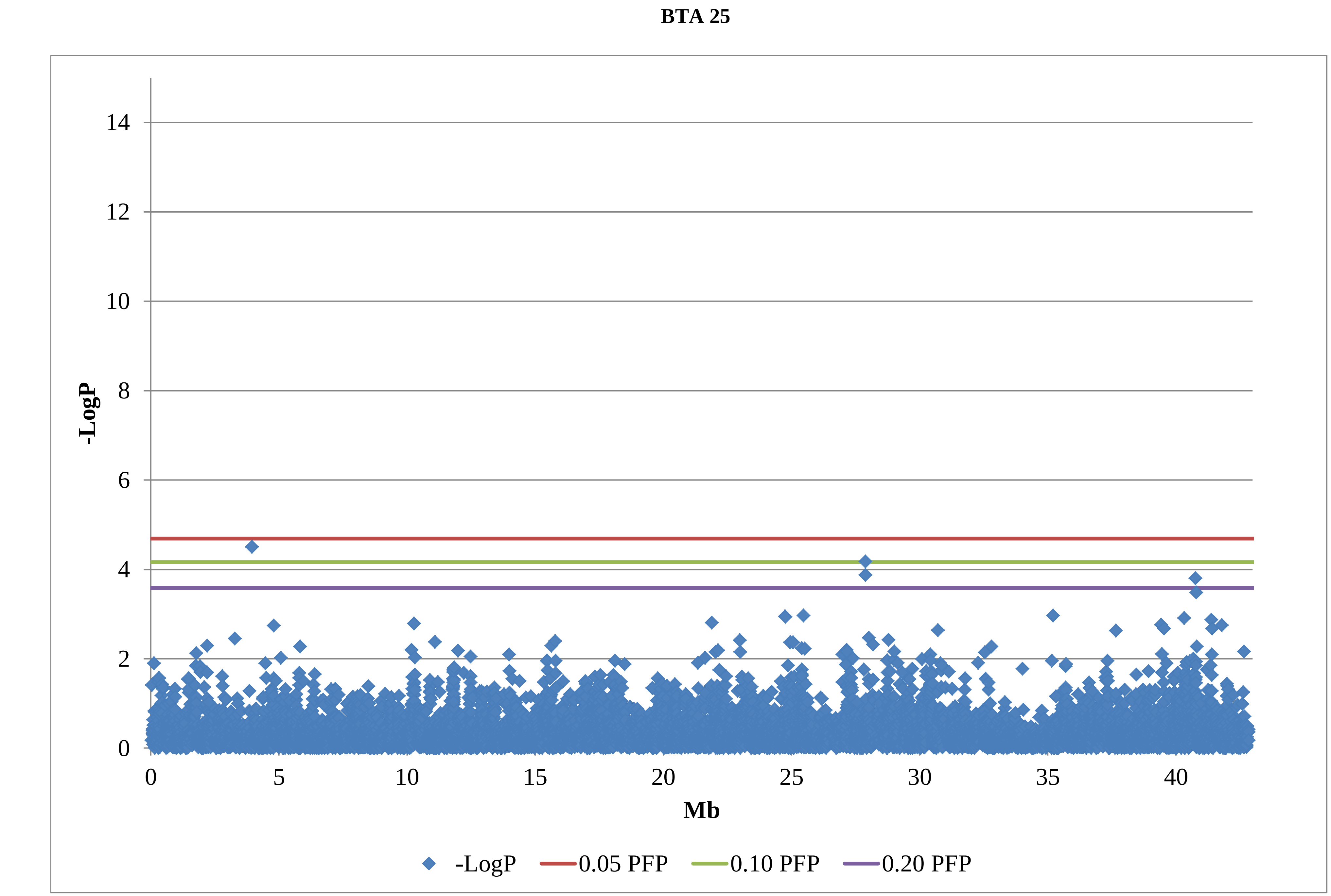
BTA 25
-LogP
14
12
10
8
6
4
2
0
0
5
10
15
20
25
30
35
40
Mb
-LogP
0.05 PFP
0.10 PFP
0.20 PFP

BTA 26

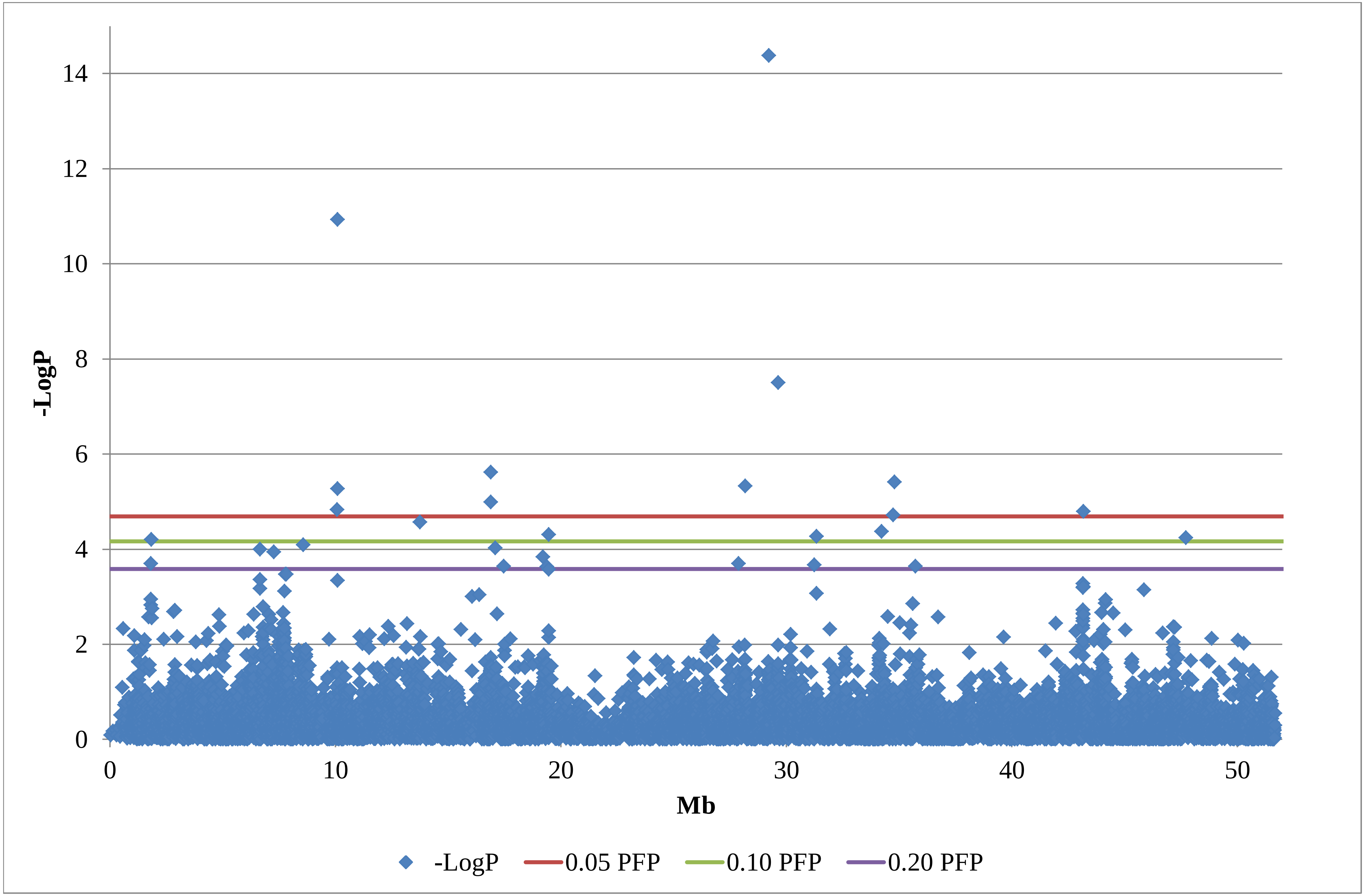

-LogP
14
12
10
8
6
4
2
0
0
10
20
30
40
50
Mb
-LogP
0.05 PFP
0.10 PFP
0.20 PFP

BTA 27

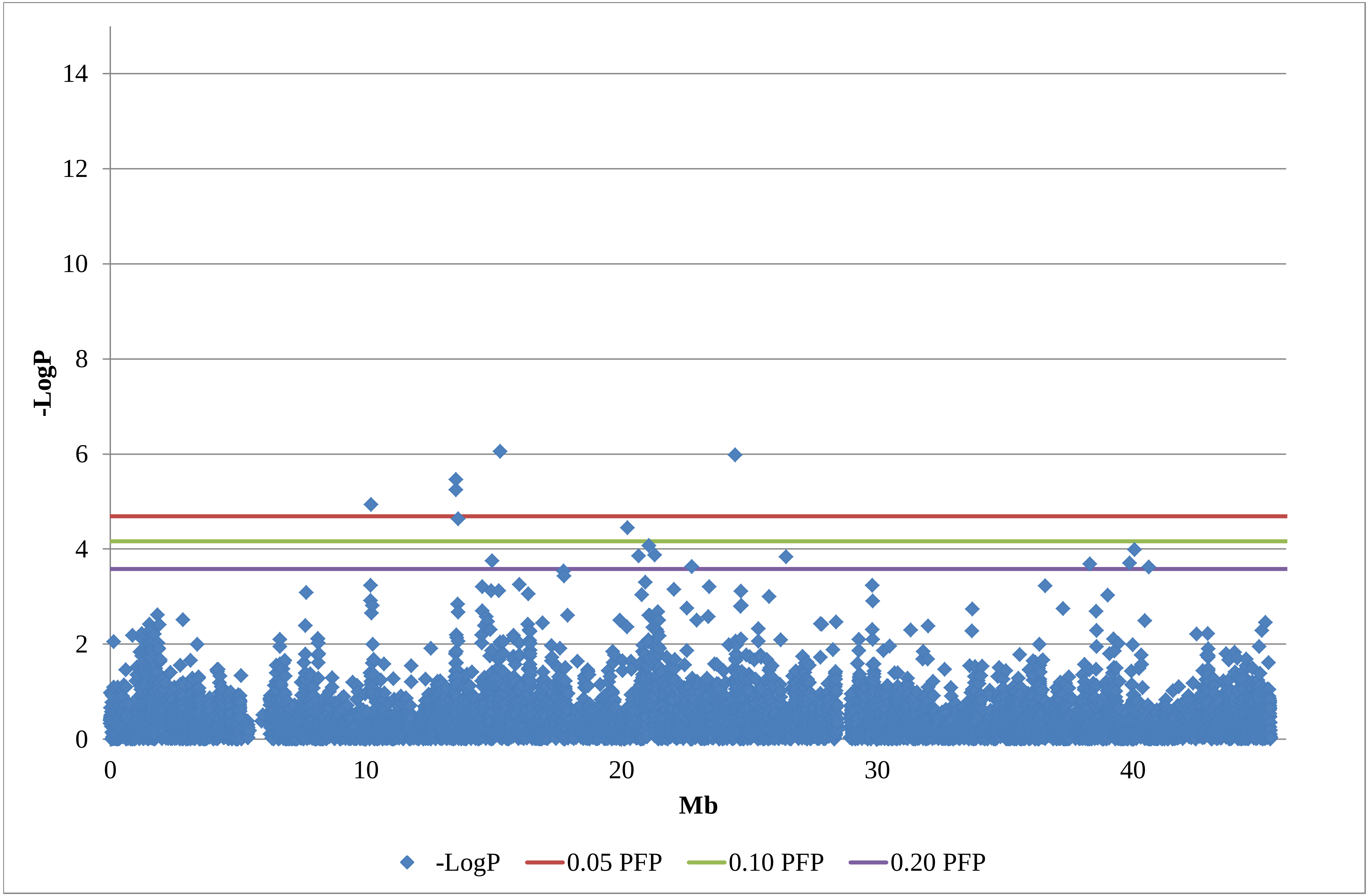

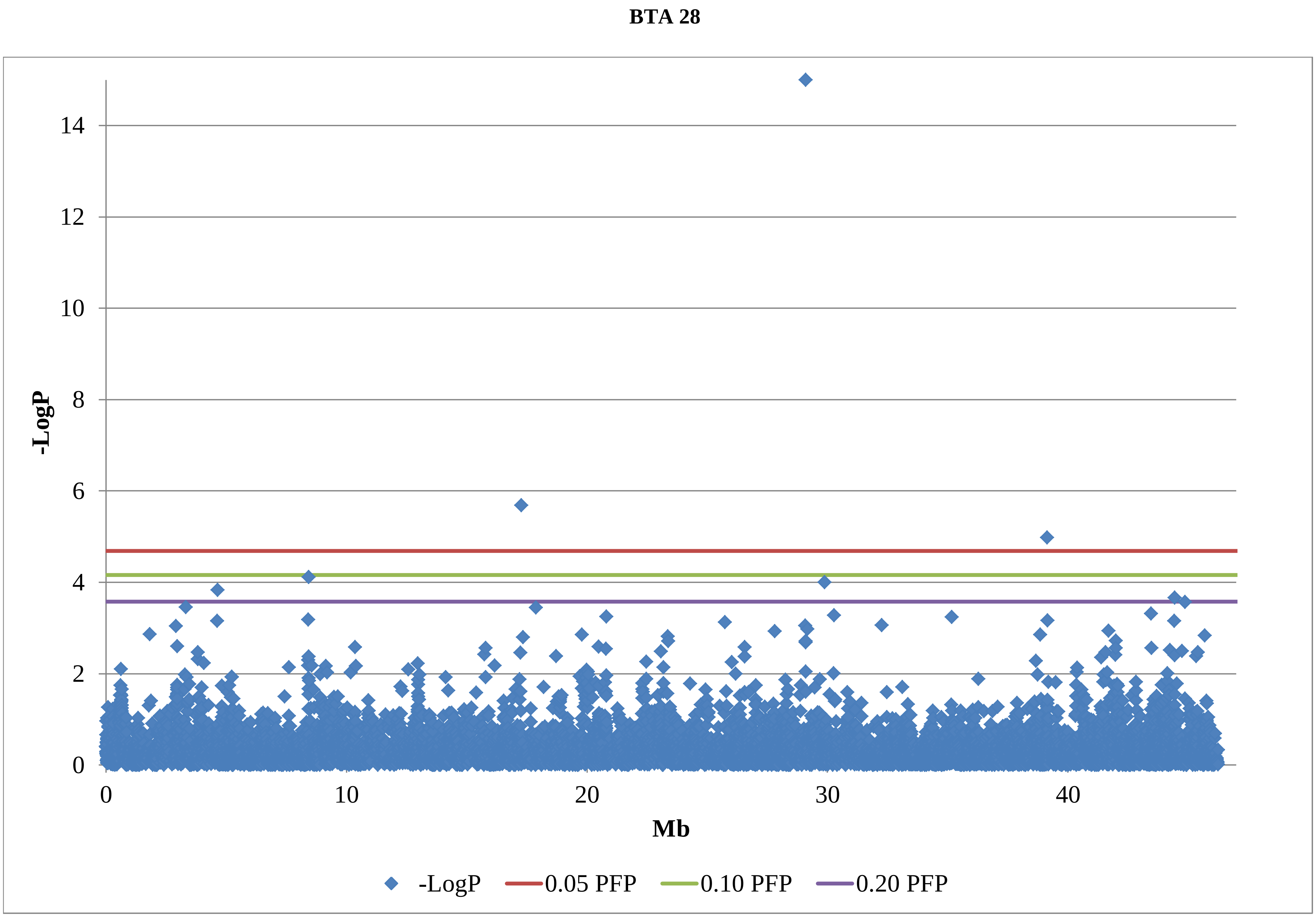

BRD pools -logP chr e.xlsx

BTA 29

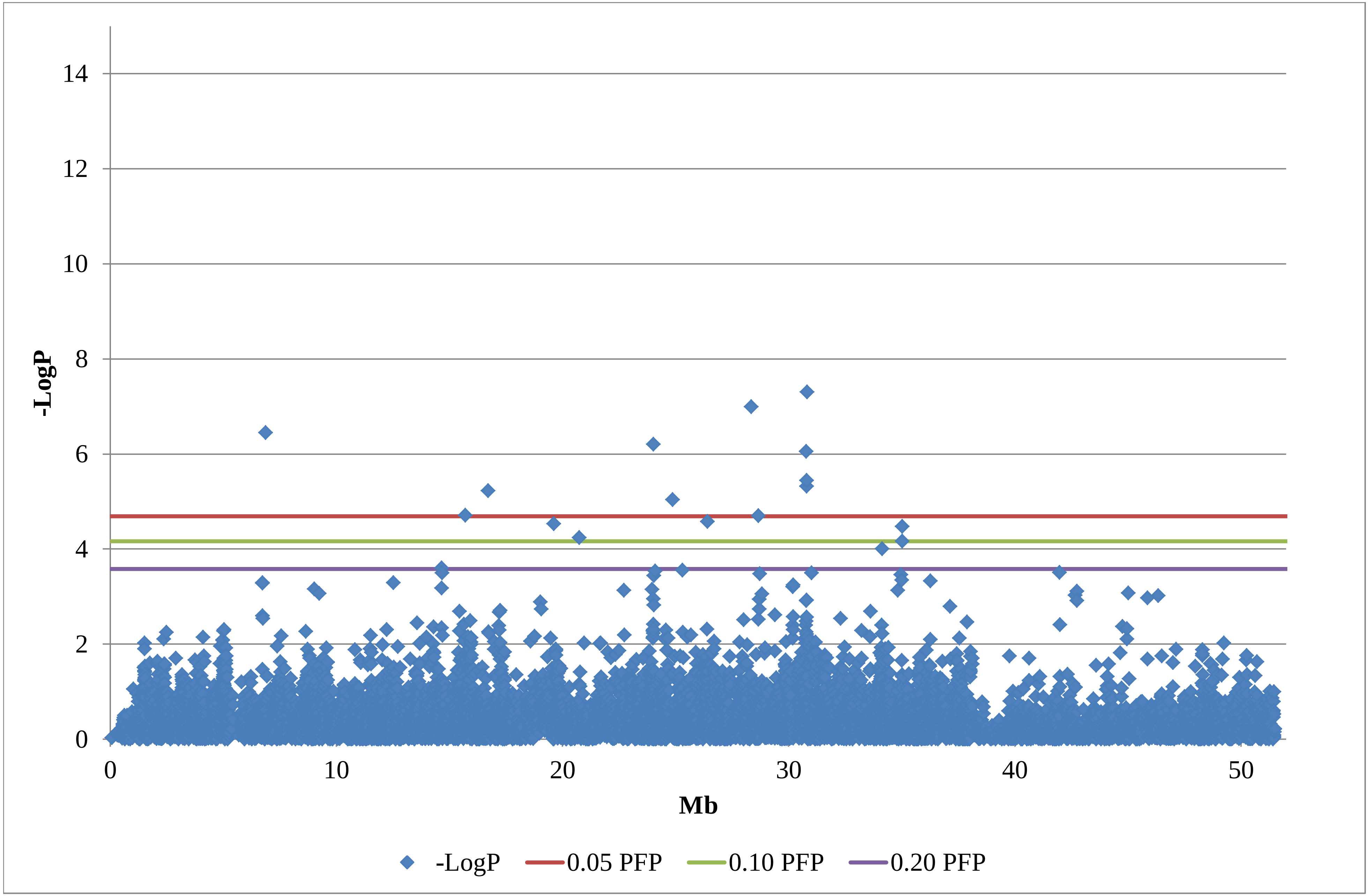

14
12
10
8
6
4
2
0
-LogP
0
10
20
30
40
50
Mb
-LogP
0.05 PFP
0.10 PFP
0.20 PFP
